# Unraveling the Contribution of High Temperature Stage to Jiang-Flavor Daqu, a Liquor Starter for Production of Chinese Jiang-Flavor Baijiu, With Special Reference to Metatranscriptomics

**DOI:** 10.3389/fmicb.2019.00472

**Published:** 2019-03-12

**Authors:** Zhuolin Yi, Yanling Jin, Yao Xiao, Lanchai Chen, Li Tan, Anping Du, Kaize He, Dayu Liu, Huibo Luo, Yang Fang, Hai Zhao

**Affiliations:** ^1^College of Pharmacy and Biological Engineering, Chengdu University, Chengdu, China; ^2^Key Laboratory of Environmental and Applied Microbiology, Chengdu Institute of Biology, Chinese Academy of Sciences, Chengdu, China; ^3^Environmental Microbiology Key Laboratory of Sichuan Province, Chengdu, China; ^4^Analytical and Testing Center, Sichuan University of Science and Engineering, Zigong, China; ^5^Key Laboratory of Bio-Resources and Eco-Environment of Ministry of Education, College of Life Sciences, Sichuan University, Chengdu, China; ^6^Bioengineering College, Sichuan University of Science and Engineering, Zigong, China

**Keywords:** flavor generation, Chinese baijiu, metatranscriptomics, Jiang-flavor daqu, saccharification, high temperature stage, degradation of aromatic compounds

## Abstract

Jiang-flavor (JF) daqu is a liquor starter used for production of JF baijiu, a well-known distilled liquor in China. Although a high temperature stage (70°C) is necessary for qualifying JF daqu, little is known regarding its active microbial community and functional enzymes, along with its role in generating flavor precursors for JF baijiu aroma. In this investigation, based on metatranscriptomics, fungi, such as *Aspergillus* and *Penicillium*, were identified as the most active microbial members and 230 carbohydrate-active enzymes were identified as potential saccharifying enzymes at 70°C of JF daqu. Notably, most of enzymes in identified carbohydrate and energy pathways showed lower expression levels at 70°C of JF daqu than those at the high temperature stage (62°C) of Nong-flavor (NF) daqu, indicating lowering capacities of saccharification and fermentation by high temperature stage. Moreover, many enzymes, especially those related to the degradation of aromatic compounds, were only detected with low expression levels at 70°C of JF daqu albeit not at 62°C of NF daqu, indicating enhancing capacities of generating special trace aroma compounds in JF daqu by high temperature stage. Additionally, most of enzymes related to those capacities were highly expressed at 70°C by fungal genus of *Aspergillus*, *Coccidioides*, *Paracoccidioides*, *Penicillium*, and *Rasamsonia*. Therefore, this study not only sheds light on the crucial functions of high temperature stage but also paves the way to improve the quality of JF baijiu and provide active community and functional enzymes for other fermentation industries.

## Introduction

**FIGURE 1:**
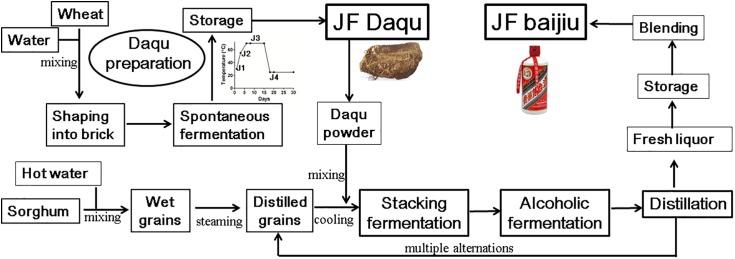
Process diagram of traditional production of Jiang-flavor (JF) baijiu. JF daqu was produced with two steps of spontaneous fermentation in a Qu-room and drying without ventilation in a storage room, and its maximum cultural temperature was usually between 60 and 70°C. Besides stage of daqu preparation, JF baijiu was produced with other three distinct stages of stacking fermentation, multiple alternations of alcoholic fermentation and distillation processes.

Baijiu (Chinese liquor), one of the oldest known distilled liquors with an approximate 2000-year history, is the largest consumed spirit globally (over 13 billion liters in 2016) (Liu and Sun, 2018). Compared with whisky and brandy, baijiu is well known for its taste with more flavor compounds (>1870 volatile compounds) in liquor, including alcohols, aldehydes, organic acids, esters, phenols, lactones, heterocycles, terpenes, aromatic compounds, amino acids, and peptides, which leads to the final special aroma and health of baijiu (Jin et al., 2017; Liu and Sun, 2018). Thus, based on its distinctive flavor characteristics, baijiu can be divided into three major categories [i.e., Jiang-flavor (JF, also called sauce-flavor) baijiu, Nong-flavor (NF) baijiu and Qing-flavor (QF) baijiu] and nine minor categories, among which JF baijiu is with a full-bodied long-lasting aroma (Zheng and Han, 2016; Liu and Sun, 2018). The representative JF baijiu is moutai, the most famous baijiu, having the distinction as “the national liquor” and largely dominating the market in China (Zheng and Han, 2016; Jin et al., 2017). JF baijiu is fermented and distilled under solid-state conditions with a production process that mainly includes four distinct stages; i.e., daqu preparation (approximately 4 months), stacking fermentation (2–4 days), alcoholic fermentation and distillation processes (Figure 1) (Fan et al., 2012; Zheng and Han, 2016).

Typically, daqu, a liquor starter used to initiate the alcoholic fermentation process, constitutes the most essential component for alcoholic fermentation, not only providing the microbial community and enzymes (as a saccharifying and fermenting agent) for alcoholic fermentation but also significantly contributing to the final liquor flavor (Zheng and Han, 2016; Liu and Sun, 2018). Similar liquor starters can be found in many Asian countries, e.g., xiaoqu/fuqu in China (Zheng and Han, 2016; Jin et al., 2017), meju in Korea (Kim et al., 2011), ragi in Indonesia (Fibri and Frøst, 2019), marcha/thiat/dawdim/hamei/chowan in India (Sha et al., 2018), bubod in Philippines (Tamang et al., 2016), and banh men in Vietnam (Thanh et al., 2008). All those liquor starters are prepared in an open system with starchy materials (wheat, rice, etc), shaped into different sizes and shapes, and cultured under different conditions (temperature and time) (Liu and Sun, 2018; Sha et al., 2018; Waché et al., 2018). Among them, JF daqu is made from wheat, shaped into brick, and produced with two steps of spontaneous fermentation for approximately 1 month in a Qu-room and drying for another 3 months without ventilation in a storage room (Huang et al., 2017b; Jin et al., 2017). During the spontaneous fermentation process, the cultivation of JF daqu is controlled by manually turning over the bricks and opening/closing the windows to change the ventilation and temperature, with the special microbial community being enriched from raw materials and the working environments by environmental variables (temperature and moisture), among which temperature serves as a key driving force (Huang et al., 2017a; Xiao et al., 2017; Liu and Sun, 2018). According to the maximum temperature in the daqu preparation process, JF daqu is grouped into high-temperature (60–70°C) daqu and requires cultivation at the high temperature stage for approximately 7–8 days (Huang et al., 2017b; Liu and Sun, 2018). Owing to this high temperature stage, the thermophilic microbial community may be enriched in JF daqu and various thermostable enzymes (i.e., proteinase, glucoamylase, cellulase, alpha-amylase, and esterase) may also be produced to degrade materials and generate special flavor compounds.

Recently, the daqu microbial community has been studied throughout fermentation by culture-dependent and -independent methods, and their diversity and dynamics are well understood (Yan et al., 2013; Wang and Xu, 2015; Huang et al., 2017b; Xiao et al., 2017). However, little is known regarding the active microbial community and their metabolic functions. In addition, although numerous crude enzymes have been identified in daqu (Li et al., 2015; Liu et al., 2018), active enzymes and their relationships with the microbial community are yet unknown. Metatranscriptomics constitutes an ideal tool for studying daqu microbial ecology, as it directly analyzes mRNA from environments and provides information not only on the microbial community composition but also on active members and their specifically expressed enzymes (Bokulich et al., 2016). This technology has been successfully applied in microbial ecological systems; e.g., compost (Mello et al., 2017), mouse gut (Just et al., 2018), cattle rumen (Pandit et al., 2018), sludge (Xia et al., 2018), ocean (Yoshida et al., 2018), and human feces (Abu-Ali et al., 2018). Nevertheless, owing to the complicated conditions in baijiu brewing systems, such as the high content of starch and fermentation products along with strongly colored materials, it remains challenging to extract high-quality RNA from baijiu fermentation samples, especially from the high temperature stage (70°C) of JF daqu, in which greater amounts of fermentation products were generated with strong colors than in all the other daqus’ making stages. Thus, to our knowledge, only samples from the JF alcoholic fermentation process (42.8°C) (Song et al., 2017) and Nong-flavor (NF) daqu (a medium-temperature daqu) preparation process (62°C) (Huang et al., 2017a) have previously been studied using metatranscriptomics.

The cultivation temperatures in the production process for JF daqu, a typical high-temperature daqu, are largely higher than those in other daqus including NF daqu (Huang et al., 2017b). The high temperature condition constitutes the most striking difference among the daqu production processes of JF daqu and other daqus, as well as their subsequent alcoholic fermentation processes, which results in unique microbial community, enzymes, and aroma compounds being generated in the JF daqu and fermented feedstock (Wu et al., 2009; Wang et al., 2014; Xiao et al., 2016; Liu and Sun, 2018). Compared with NF daqu, JF daqu has a lower capacity for saccharification, liquefaction, and fermentation (Zheng and Han, 2016; Liu and Sun, 2018), thus requiring the use of a large amount of JF daqu (nearly 1:0.9 ratio of daqu versus feedstock) in the alcoholic fermentation process, which is higher than that of NF daqu (approximately 1:2 ratio). Thus, the flavor precursors, enzymes, and microbial community enriched in JF daqu would likely be more strongly associated with the final liquor flavor than those in NF daqu. Recently, we have published breakthrough research wherein significant differences were predictively shown in energy, carbohydrate metabolism, and degradation of aromatic compounds between the JF daqu and NF daqu bacterial community (Huang et al., 2017b), and the active microbial community was found to highly express pivotal enzymes at the high temperature stage of NF daqu making process (Huang et al., 2017a). However, the active microbial community and important enzymes, as well as their functional correlations in JF daqu remain to be identified. More specific understanding regarding differences of the high temperature stage between JF and NF daqu have not been clarified. Therefore, in this study, we first employed metatranscriptomics to explain the structure and function of the actual microbial community and its pivotal enzymes at the high temperature stage of JF daqu making process. Moreover, a comprehensive and global comparison was performed between JF and NF daqu to shed light on functions of the high temperature stage with regard to saccharification and fermentation along with flavor compound generation. This study provides fundamental information related to the active microbial community and functional enzymes and may facilitate a comparative understanding of the pivotal role of the high temperature stage in the JF daqu making process and JF baijiu brewing.

## Materials and Methods

### Sample Collection

JF daqu samples were collected at different time points from a fermentation workshop of Kweichow Hanwang Group Co., Ltd. in Renhuai, Guizhou, China, as described previously (Huang et al., 2017b). Briefly, Sample J1 was harvested at the beginning of daqu production (30°C); J2 was harvested after 3 days of daqu preparation (55°C); J3 was harvested after 8 days of daqu preparation (70°C); and J4 was harvested from the mature daqu after fermentation for 20 days (25°C) (Figure 1). In addition, all samples were selected and mixed from three locations in the same Qu-room at each time point. For RNA extraction, the daqu samples were frozen in liquid nitrogen immediately after collection, transferred to the Chengdu Biology Institute, Chinese Academy of Sciences on that day and stored in a -80°C freezer. For enzyme analysis, all the samples were suspended in 0.1% (v/v) Tween 80 solution and transferred to the institute at room temperature (Huang et al., 2017a).

### Carbohydrate-Degrading Enzyme Activities

A total of 18 polymer analogs of insoluble chromogenic AZurine Cross-Linked (AZCL) polysaccharides (Megazyme, Ireland) were selected for detecting enzyme activities on cellulose, hemicellulose, starch, chitin, and glucan degradation (Table 1). As in our prior study (Huang et al., 2017a), all daqu samples in 0.1% (v/v) Tween 80 solution were incubated at 25°C and 100 rpm overnight, then their supernatants were added directly onto the wells of solid plates with AZCL polysaccharides according to the manufacturer’s protocol. After incubation at 35, 45, or 55°C for 22 h, carbohydrate-degrading enzyme activities were determined by measuring the diameter of the blue haloes, which were recorded in millimeters.

**Table 1:** Relative abundances of highly active fungal and bacterial taxa according to their designated gene numbers by the NR database.

			Relative
Classification			abundance (%)
Fungi			97.7
	*Aspergillus*		53.2
		*Neosartorya fischeri*	9.9
		*Aspergillus oryzae*	9.9
		*Aspergillus fumigatus*	7.7
		*Aspergillus niger*	6.3
		*Aspergillus clavatus*	5.8
		*Aspergillus terreus*	5.4
		*Aspergillus kawachii*	3.9
		*Aspergillus nidulans*	2.3
		*Aspergillus flavus*	2.1
	*Penicillium*		29.2
		*Penicillium stipitatus*	14.7
		*Penicillium marneffei*	11.1
		*Penicillium chrysogenum*	2.2
		*Penicillium digitatum*	1.2
	*Ajellomyces*		3.6
		*Ajellomyces dermatitidis*	2.0
		*Ajellomyces capsulatus*	1.6
	*Coccidioides*		2.2
		*Coccidioides posadasii*	1.1
		*Coccidioides immitis*	1.1
	*Paracoccidioides*		1.4
		*Paracoccidioides brasiliensis*	1.4
	*Uncinocarpus*		0.9
		*Uncinocarpus reesii*	0.9
	*Arthroderma*		0.7
	*Exophiala*		0.6
		*Exophiala dermatitidis*	0.6
	*Trichophyton*		0.6
	*Macrophomina*		0.5
		*Macrophomina phaseolina*	0.5
Yeast			0.2
Bacteria			2.1
	*Saccharopolyspora*		0.6
	*Acinetobacter*		0.3
	*Kurthia*		0.2


### RNA Extraction and Sequencing

Similar to the RNA extraction from NF daqu (Huang et al., 2017a), total RNA was extracted from JF daqu samples using borate buffer, cleaned with the RNeasy Midi Kit (Qiagen #75142, Venlo, Netherlands) and treated with DNase I (Fermentas, Waltham, MA, United States) according to the manufacturer’s protocols. The RNA integrity was evaluated by gel electrophoresis and RNA integrity number (RIN) was checked using an Agilent2100 Bioanalyzer (Santa Clara, CA, United States). RNA samples with RIN value greater than 7.0 and OD260/OD280 ratio greater than 1.8 were selected for deep sequencing.

Total RNA (approximately 20 μg) from J3 was used for the RNA sequencing. Prior to metatranscriptomic library construction, using a previously reported method (Huang et al., 2017a), mRNA was isolated using magnetic beads with Oligo (dT) for eukaryotes, and for prokaryotes, mRNA was obtained after removing ribosomal RNA. The isolated mRNA was first fragmented and then used as template for subsequent first- and second-strand cDNA synthesis with random primers. Short cDNA fragments were purified and resolved with EB buffer for end reparation and poly(A) addition. Thereafter, the short cDNA fragments were ligated to sequencing adapters and suitable sized cDNA fragments were purified as templates for polymerase chain reaction amplification. RNA sequencing of the library was performed using platform (Illumina, San Diego, CA, United States) at the Beijing Genomics Institute (BGI)- the HiSeqTM 2000 Shenzhen, China.

### Metatranscriptomics Assembly and Annotation

As for our previous metatranscriptomics assembly of NF daqu samples (N1–4), raw sequenced reads of J3 were first filtered by removing adaptors, low quality reads, and the rRNA sequences (Li et al., 2009). The clean reads of J3 were then *de novo* assembled using Trinity^1^ (Grabherr et al., 2011), by which unigene sequences were generated. To annotate the metatranscriptome, the unigene sequences were aligned using Blastx (version 2.5.0) with protein and nucleotide databases including Non-redundant protein (NR), Non-redundant nucleotide (NT), Swiss-Prot, Kyoto Encyclopedia of Genes and Genomes (KEGG), Clusters of Orthologous Groups (COG), and Gene Ontology (GO) (*e*-value < 10^-5^), and identified according to the highest similarity to known sequence. In cases of the non-alignment of unigenes against one of the listed databases, ESTScan was used to determine their coding directions. Thereafter, according to the standard codon usage, coding DNA sequences (CDSs) were translated into protein sequences. KEGG pathways were extracted from the KEGG web server^2^ (Kanehisa et al., 2017). WEGO software^3^ was used for GO classification (Ye et al., 2006). Carbohydrate-active enzymes (CAZymes) were retrieved from the Carbohydrate-Active Enzymes database (CAZy)^4^ (Lombard et al., 2014).

### Identification of Differentially Expressed Genes (DEGs) and Pathway Analysis

To compare the gene expression levels among J3 and NF daqu samples (N1–4), the predicted ORFs were combined after removing redundancy using cd-hit (Version 4.6.1)^5^ (Li and Godzik, 2006). Gene expression levels were calculated using the Reads Per Kilobase per Million mapped reads (RPKM) method (Mortazavi et al., 2008). DEGs among J3 and NF daqu samples were identified using a method based on the Poisson distribution (Audic and Claverie, 1997). DEGs between two samples were identified using *p*-value ≤ 0.05, Log_2_(RPKM ratio) ≥ 1, and false discovery rate (FDR) value ≤ 0.001 (Benjamini and Yekutieli, 2001). To analyze GO enrichment, all DEGs were mapped to terms of the GO database.

### Accession Number

The raw and assembled metatranscriptomics data of J3 have been deposited to the GenBank database under accession numbers SRR7785758 and GGWC00000000, respectively.

## Results

### RNA Sequencing and Metatranscriptomics Assembly

After RNA sequencing of the J3 sample, 5.882 Gbp of raw data was generated, from which 5.663 Gbp of clean data was then obtained by filtering (Supplementary Table S1). These clean data were *de novo* assembled, from which 38,899 unigenes were identified with a total length of 46,187,298 nucleotides (nt) and N50 length of 2232 bp (Supplementary Table S2). As shown in Supplementary Figure S1, there were 3585 unigenes with sequence size > 3000 nt.

### Functional Annotation and Classification of Unigenes

To annotate the unigenes of J3, blastx alignment against the protein and nucleotide databases of NR, NT, Swiss-Prot, KEGG COG and GO was performed; the results are shown in Supplementary Table S3. The CDSs that mapped to the protein database and were predicted by ESTscan numbered 30,615 and 1041, respectively. A total of 31,279 known unigenes were identified by blastx, among which 14,912 genes were annotated by COG classification. There were 25 classes in the COG classification with the largest number of unigenes being found solely in the class of “general function prediction” (15.1%; Supplementary Figure S2). In addition, 19,468 unigenes were also annotated by the GO database, which accounted for 50.1% of all the unigenes, with the annotations grouped into three categories (biological process; cellular component; and molecular function) (Supplementary Figure S3). “Metabolic processes,” “cell” and “catalytic activities” were dominant in the categories of biological processes, cellular components, and molecular functions, respectively.

Overall, 30,793 genes (*e*-value < 10^-5^) were annotated using the NR database (Supplementary Table S3), which is far higher than those by other databases; the composition of active bacterial and fungal taxa in J3 is presented in Table 1. Based on their gene numbers, the active fungal community was more prevalent than the bacterial community and accounted for 97.7% in J3. In the fungal component, *Aspergillus* and *Penicillium* were the pivotal genera with high relative abundances of 53.2 and 29.2%, respectively. In addition, the active yeast showed low relative abundances of 0.2% at this high temperature stage.

**FIGURE 2:**
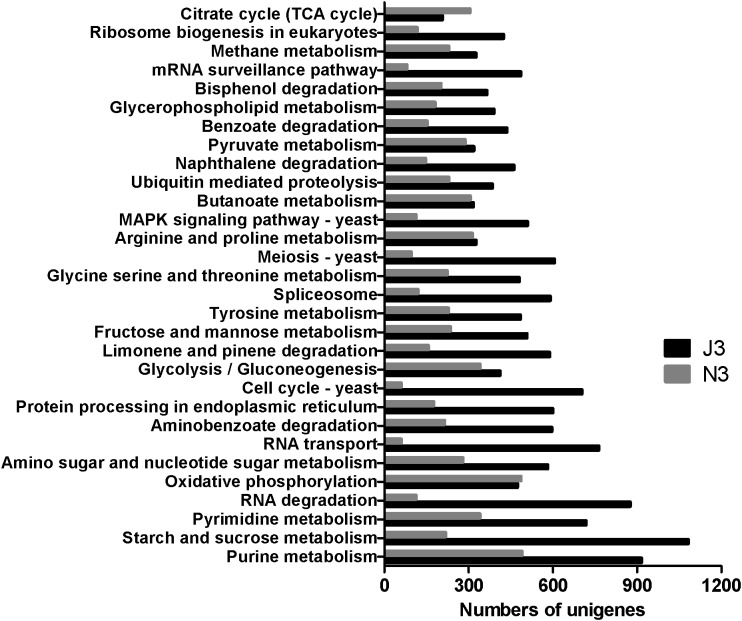
The 30 most abundant KEGG pathways in high temperature stage samples of J3 and N3. J3 was harvested after 8 days of JF daqu preparation and N3 was harvested after 9 days of NF daqu preparation. The temperatures of J3 and N3 were 70 and 62°C, respectively.

As shown in Figure 2, starch and sucrose metabolism had the highest number of unigenes in J3, and except for the citrate cycle (TCA cycle) and oxidative phosphorylation, all of the 30 most abundant KEGG pathways showed higher numbers of unigenes in J3 than those in the high temperature stage (N3) of NF daqu. Moreover, large differences were found between J3 and N3 in basic metabolisms (i.e., purine metabolism, RNA degradation, RNA transport, meiosis-yeast, MAPK signal pathway-yeast, cell cycle-yeast, spliceosome, and mRNA surveillance pathway), degradation of aromatic compounds (aminobenzoate degradation, naphthalene degradation, benzoate degradation and bisphenol degradation), starch and sucrose metabolism, and amino sugar and nucleotide sugar metabolism. Conversely, comparable numbers of unigenes were found between J3 and N3 in oxidative phosphorylation, glycolysis/gluconeogenesis, butanoate metabolism, and pyruvate metabolism. Additionally, metabolism of amino acids; i.e., tyrosine, glycine, serine, threonine, arginine, and proline, were also ranked in the top 30 of both J3 and N3.

### Identification of CAZymes

**FIGURE 3:**
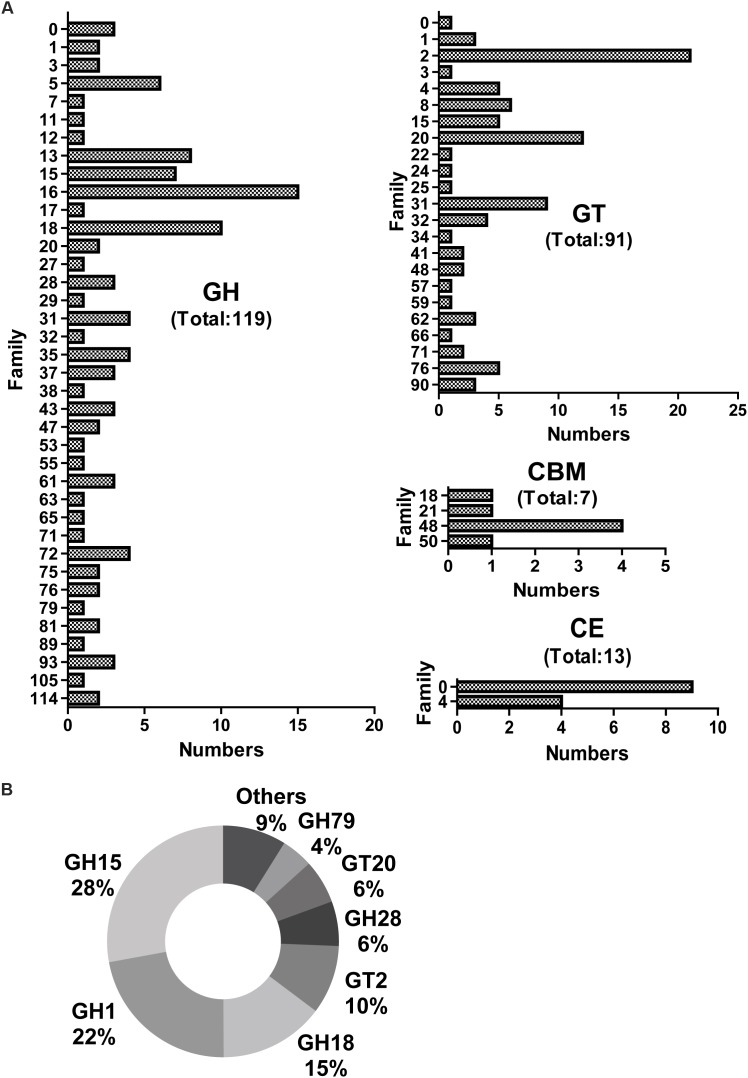
Numbers and expression levels of carbohydrate-active enzymes in J3. Matched unigenes of carbohydrate-active enzymes are shown with numbers **(A)** and expression levels **(B)**. GH, glycoside hydrolase; GT, glycosyl transferase; CBM, carbohydrate-binding module; and CE, carbohydrate esterase.

Screening for genes encoding putative CAZymes from the metatranscriptomic library of J3 identified glycoside hydrolases (GH) (119) and glycosyl transferases (GT) (91) as having higher numbers of unigenes than carbohydrate esterases (CE) (13) and carbohydrate-binding modules (CBM) (7) (Figure 3A). Additionally, the total number (230) of these CAZymes in J3 was lower than that (932) in the N3 sample of NF daqu (Huang et al., 2017a). The expression level for all CAZymes in J3 showed a total RPKM value of 460.8, which was markedly lower than that of 20789.9 in N3 (Supplementary Table S4). The CAZyme classes with relatively high expression levels in J3 comprised GH15 (28%), GH1 (22%), GH18 (15%), GT2 (10%), GH28 (6%), GT20 (6%), and GH79 (4%), which totally differed from those in N3 (Figure 3B and Supplementary Table S4). These major GH families may have activities of glucoamylase (GH15), beta-glucosidase (GH1), chitinase (GH18), cellulose synthase and chitin synthase (GT2), polygalacturonase (GH28), alpha, alpha-trehalose phosphate synthase (GT20), and β-glucuronidase (GH79).

Moreover, insoluble chromogenic AZCL polysaccharide assays at different reaction temperatures (35, 45, and 55°C), clearly detected only endo-β-1,3-1,4-glucanase, endo-1,4- β -D-galactanase, and rhamnogalacturonanase as exhibiting activity at the high temperature stage of J3 (Table 2), which to some extent was complementary to the metatranscriptic results. In comparison, a broad spectrum of CAZymes was detected in the initial sage (J1) and mature stage (J4), and four CAZymes were also found in the high temperature stage of J2. Notably, only one CAZyme of α-amylase with thermophilic activity was obviously present, showing higher activity at higher temperatures in J1, whereas more CAZymes were clearly found with thermophilic activities in J2, J3, and J4, such as α-amylase, endo-β-1,3-1,4-glucanase, endo-proteases, and endo-1,4- β -D-xylanase.

**Table 2:** Carbohydrate-active enzyme analysis of Jiang-flavor daqu.

Substrate	Enzyme	Diameter (mm)			
		
		J1	J2	J3	J4
		35/45/55°C	35/45/55°C	35/45/55°C	35/45/55°C
AZCL-curdlan	Endo-1,3- β -D-glucanase	15/13.5/7	7/3/6		
AZCL-beta-glucan	Endo-β-1,3-1,4-glucanase	6/9/7	0.5/0/2	7/8/13	1/5.5/11
AZCL-he-cellulose	Endo-β-1,4-glucanase				
AZCL-dextran	Endo-1,6-α-D-glucanase				
AZCL-xyloglucan	Endo-β-1,4-xyloglucanase				
AZCL-amylose	α-amylase	16.5/18/20			13/14/16
AZCL-casein	Endo-proteases		0.5/2/3		6/6/8
AZCL-collagen	Endo-proteases				
AZCL-debranched arabinan	Endo-1,5-α-L-arabinanase	6/6/6			6/6/5
AZCL-galactomannan	Endo-1,4- β -D-mannanase	4/3/3			
AZCL-galactan	Endo-1,4- β -D-galactanase	5/5.5/2		0/2/1	6/6/7
AZCL-rhmnogalacturonan I	Rhamnogalacturonanase	3/5/5	3/4/5	2/4/5	9/10/8
AZCL-chitosan	Chitosanase				
AZCL-pullan	Microbial pullulanase				
AZCL-xylan	Endo-1,4- β -D-xylanase				12/15.5/17
AZCL-arabinoxylan	Endo-1,4- β -D-xylanase				15/16/18


### DEGs Among J3 and NF Daqu Samples

**FIGURE 4:**
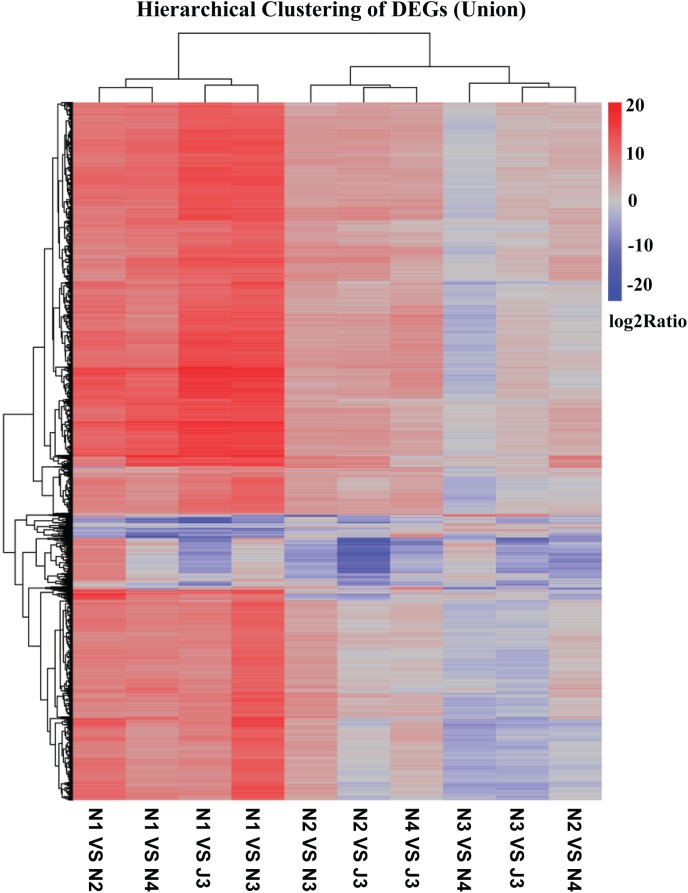
Hierarchical clustering of differentially expressed genes (DEGs) (Union) among J3 and NF samples (N1, N2, N3, and N4). DEGs between two samples were identified using *p*-value ≤ 0.05, Log_2_(RPKM ratio) ≥ 1, and false discovery rate (FDR) value ≤ 0.001.

The DEGs between the J3 and NF daqu samples were identified and a heatmap of hierarchical clustering of DEGs was constructed using log_2_(RPKM ratio) to visualize the respective patterns of DEGs. As shown in Figure 4, numerous DEGs (union) in J3, N2, N3, and N4 were clearly up-regulated with high log_2_(rations) values when compared with N1; thus, J3 together with N2–4 exhibit the largest differences in DEGs compared with N1. Alternatively, J3 presented the smallest differences in DEGs with N3. Similar results among J3 and NF daqu samples were also observed by analysis of hierarchical clustering of inter DEGs (Supplementary Figure S4). Furthermore, a comprehensive comparison performed between J3 and N3 identified a total of 14,149 unigenes as significant DEGs including 506 up- and 13,642 down-regulated genes (Supplementary Figure S5). In addition, for the GO functional classification (J3/N3), numerous DEGs were grouped into four dominant categories: “cellular processes,” “metabolic processes,” “binding,” and “catalytic activities” (Supplementary Figure S6).

### Pathway Comparisons of Starch and Sucrose Metabolism, Glycolysis, Pyruvate Metabolism, and the Citrate Cycle Between J3 and N3

**FIGURE 5:**
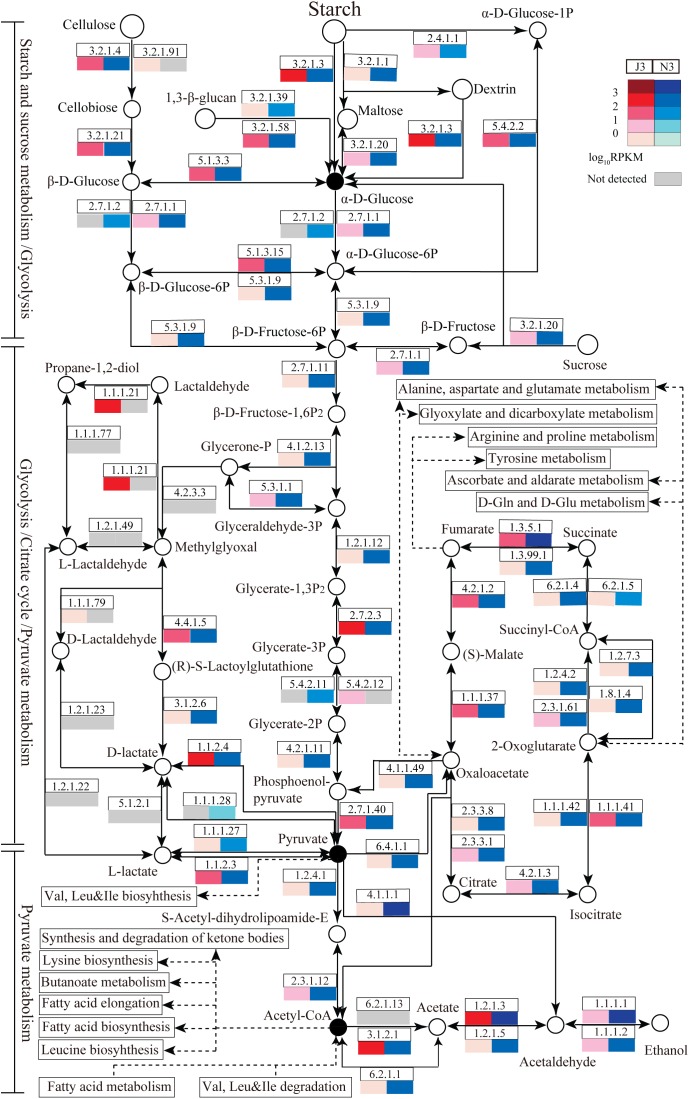
Relative abundances of enzymes related to carbohydrate and energy metabolisms in J3 and N3. Four abundant carbohydrate and energy metabolisms were analyzed including starch and sucrose metabolism, glycolysis, pyruvate metabolism, and the citrate cycle. In these pathways, enzymes with relatively high expression levels are partly presented by EC number and total RPKM. Relative expression [log_10_RPKM)] is shown in red for J3 and blue for N3. The key products are highlighted with black closed circles.

For further functional comparison of DEGs between J3 and N3, metabolic pathways were analyzed based on the KEGG database. Moreover, several key carbohydrate and energy metabolisms that were associated with relatively high numbers of unigenes were selected for comparative analysis including starch and sucrose metabolism, glycolysis, pyruvate metabolism, and citrate cycle pathways. As shown in Figure 5, enzymes related to these selected pathways were mainly present, and a complete metabolic process of converting polymers into end-products was apparent in both J3 and N3. In addition, the majority of enzymes in these four key pathways exhibited lower expression levels in J3 than in N3, with the exception of e.g., aldehyde reductase (1.1.1.21), polygalacturonase (3.2.1.15), beta-fructofuranosidase (3.2.1.26), 4-alpha-glucanotransferase (2.4.1.25), 1,4-beta-cellobiosidase (3.2.1.91), and 2,3-bisphosphoglycerate-independent phosphoglycerate mutase (5.4.2.12) (Figure 5 and Supplementary Tables S5–S8).

According to KEGG annotation, in starch and sucrose metabolism, glucoamylase (3.2.1.3), glucan 1,3-beta-glucosidase (3.2.1.58), endoglucanase (3.2.1.4), and beta-glucosidase (3.2.1.21) showed relatively high expression levels with RPKM values of 172.9, 70.1, 19.0, and 13.7, respectively, in J3; these are responsible for degrading polymers of starch, dextrin, 1,3-beta-glucan, and cellulose into glucose (Figure 5 and Supplementary Table S5). Moreover, phosphoglucomutase (5.4.2.2), which efficiently collaborates with starch phosphorylase (2.4.1.1) in converting starch into glucose-6P, also exhibited relatively high expression abundance with an RPKM value of 83.6 in J3. Additionally, most types of these enzymes were highly expressed by fungal species; e.g., *Rasamsonia emersonii*, *Aspergillus fumigatus*, *Coccidioides immitis*, and *Aspergillus oryzae* (Table 3). In comparison, some enzymes showed very low expression levels in J3, such as xylan 1,4-beta-xylosidase, alpha-amylase, and alpha-glucosidase, which to some extent was consistent with their low activities in AZCL polysaccharides assays of J3 (Table 2 and Supplementary Table S5).

**Table 3:** The top 20 expressed enzymes in starch and sucrose metabolism, glycolysis, pyruvate metabolism, and the citrate cycle in J3.

Gene ID	K0 ID	EC ID	Definition	RPKM	Species	Pathways
J3_2021	K01178	3.2.1.3	Glucoamylase	117.0	*Rasamsonia emersonii*	Starch and sucrose metabolism
J3_2988	K01835	5.4.2.2	Phosphoglucomutase	83.2	*Aspergillus fumigatus*	Starch and sucrose metabolism, Glycolysis
J3_3775	K01210	3.2.1.58	Glucan 1,3-beta-glucosidase	60.9	*Coccidioides immitis*	Starch and sucrose metabolism
J3_3326	K01178	3.2.1.3	Glucoamylase	29.4	*Aspergillus oryzae*	Starch and sucrose metabolism
J3_382	K01184	3.2.1.15	Polygalacturonase	28.0	*Aspergillus fumigatus*	Starch and sucrose metabolism
J3_2314	K00697	2.4.1.15	Alpha, alpha-trehalose phosphate synthase	26.6	*Aspergillus oryzae*	Starch and sucrose metabolism
J3_1666	K00128	1.2.1.3	Aldehyde dehydrogenase (NAD+)	514.9	*Paracoccidioides sp.*	Glycolysis, Pyruvate metabolism
J3_668	K00927	2.7.2.3	Phosphoglycerate kinase	134.7	*Penicillium stipitatus*	Glycolysis
J3_223	K00873	2.7.1.40	Pyruvate kinase	68.9	*Aspergillus fumigatus*	Glycolysis, Pyruvate metabolism
J3_870	K01785	5.1.3.3	Aldose 1-epimerase	18.3	*Aspergillus clavatus*	Glycolysis
J3_2941	K00102	1.1.2.4	D-lactate dehydrogenase (cytochrome)	141.2	*Penicillium marneffei*	Pyruvate metabolism
J3_2495	K01067	3.1.2.1	Acetyl-CoA hydrolase	134.6	*Aspergillus clavatus*	Pyruvate metabolism
J3_701	K00011	1.1.1.21	Aldehyde reductase	101.4	*Aspergillus oryzae*	Pyruvate metabolism
J3_3373	K01759	4.4.1.5	Lactoylglutathione lyase	63.2	*Neosartorya fischeri*	Pyruvate metabolism
J3_10280	K00026	1.1.1.37	Malate dehydrogenase	27.3	*Aspergillus oryzae*	Citrate cycle, Pyruvate metabolism
J3_1923	K00102	1.1.2.4	D-lactate dehydrogenase (cytochrome)	16.3	*Trichophyton rubrum*	Pyruvate metabolism
J3_909	K01679	4.2.1.2	Fumarate hydratase, class II	81.3	*Aspergillus oryzae*	Citrate cycle
J3_1885	K00235	1.3.5.1	Succinate dehydrogenase (ubiquinone) iron-sulfur subunit	62.4	*Penicillium stipitatus*	Citrate cycle
J3_1073	K00030	1.1.1.41	Isocitrate dehydrogenase (NAD+)	54.1	*Aspergillus clavatus*	Citrate cycle
J3_655	K00030	1.1.1.41	Isocitrate dehydrogenase (NAD+)	39.0	*Aspergillus terreus*	Citrate cycle


In glycolysis and pyruvate metabolisms, J3 contained an integral serial of enzymes for converting glucose into the important product, pyruvate, which would then be reversibly converted to acetyl-coA under aerobic conditions (Figure 5). Hexokinase (2.7.1.1), 6-phosphofructokinase (2.7.1.11), and pyruvate kinase represent three key enzymes in glycolysis, although only pyruvate kinase (2.7.1.40) showed relatively high expression abundance (RPKM value of 75.5) in J3, which could irreversibly produce pyruvate from phosphenol-pyruvate (Figure 5 and Supplementary Tables S6, S7). Alternatively, pyruvate dehydrogenase E1 (1.2.4.1), pyruvate dehydrogenase E2 (2.3.1.12), and acetyl-CoA synthase (6.2.1.1) are responsible for producing acetyl-CoA from pyruvate and acetate, respectively; all of these showed relatively low RPKM values in J3. Moreover, aldehyde dehydrogenase (NAD+) (1.2.1.3), which reversibly produced acetate from acetaldehyde, had the highest relative expression level with an RPKM value of 515.0, and acetyl-CoA hydrolase (3.1.2.1), which irreversibly produced acetate from acetyl-CoA, was also highly expressed in glycolysis and pyruvate metabolisms of J3 (Figure 5 and Supplementary Tables S6, S7). Thus, acetaldehyde could be then converted to ethanol under anaerobic conditions by alcohol dehydrogenases (1.1.1.1 and 1.1.1.2) (Figure 5 and Supplementary Table S6), which were expressed at low levels by fungal species of *A. fumigatus*, *C. immitis*, *Aspergillus terreus*, *Coccidioides posadasii*, *Penicillium marneffei*, and *Neosartorya fischeri* in J3 (data not shown). Therefore, high concentration of acetate and low concentration of ethanol could be accumulated by collaborations of aldehyde dehydrogenase (NAD+), acetyl-CoA hydrolase, and alcohol dehydrogenases. In addition, aldehyde reductase (1.1.1.21), phosphoglycerate kinase (2.7.2.3), and lactoylglutathione lyase (4.4.1.5), which could produce lactaldehyde, phosphoglycerate, glucose, and lactoylglutathione, respectively, also showed relatively high expression levels and made large contributions in glycolysis and pyruvate metabolisms of J3. Highly expressed types of these enzymes also originated from fungal species, such as *Paracoccidioides sp.*, *Penicillium stipitatus*, *A. fumigatus*, *Aspergillus clavatus*, *Aspergillus oryzae*, and *N. fischeri* (Table 3). Considering that pyruvate and acetyl-coA serve as important intermediates for Val, Leu, and Ile biosynthesis, fatty acid biosynthesis, butanoate metabolism, leucine biosynthesis, and the synthesis and degradation of ketone bodies (Figure 5), the J3 samples thus showed capacities for converting glucose to pivotal intermediates of pyruvate and acetyl-coA for fatty acids, amino acids, and carbohydrates, which would further make large contributions for generating specific flavor in JF daqu.

In conditions of insufficient oxygen, pyruvate can be reversibly converted to lactate by L-lactate dehydrogenase (1.1.1.27), D-lactate dehydrogenase (1.1.1.28), D-lactate dehydrogenase (cytochrome) (1.1.2.4), or L-lactate dehydrogenase (cytochrome) (1.1.2.3); in particular, D-lactate dehydrogenase (cytochrome) showed relatively high expression abundance with an RPKM value of 162.1 in J3 (Figure 5 and Supplementary Table S7). Members of D-lactate dehydrogenase (cytochrome) were specifically highly expressed by *P. marneffei* and *Trichophyton rubrum* (Table 3).

Almost all of the enzymes of the citrate cycle were present in the J3 sample (Figure 5). Among these, isocitrate dehydrogenase (NAD+) (1.1.1.41), fumarate hydratase (4.2.1.2), succinate dehydrogenase (1.3.5.1), and malate dehydrogenase (1.1.1.37) showed relatively high expression abundances with RPKM values of 94.1, 81.3, 63.0, and 37.8, respectively (Supplementary Table S8). Highly expressed members of these enzymes were mostly derived from fungal species, such as *A. oryzae*, *T. stipitatus*, *A. clavatus*, and *A. terreus* (Table 3). In addition, 2-oxoglutarate dehydrogenase E2 (2.3.1.61), citrate synthase (2.3.3.1), and aconitate hydratase 1 (4.2.1.3) were also clearly detected. Moreover, ATP citrate (pro-S)-lyase (2.3.3.8), phosphoenolpyruvate carboxykinase (ATP) (4.1.1.49), and pyruvate carboxylase (6.4.1.1), which are key enzymes that connect the citrate cycle and pyruvate metabolism, were still detected in J3. As shown in Figure 5, oxaloacetate, fumarate, and 2-oxoglutarate are pivotal intermediates for alanine, aspartate, and glutamate metabolism, arginine and proline metabolism, tyrosine metabolism, and D-Gln and D-Glu metabolism, which would then also contribute to specific flavor generation in JF daqu.

### Abundant Comparisons of Enzymes for the Degradation of Aromatic Compounds Between J3 and N3

**FIGURE 6:**
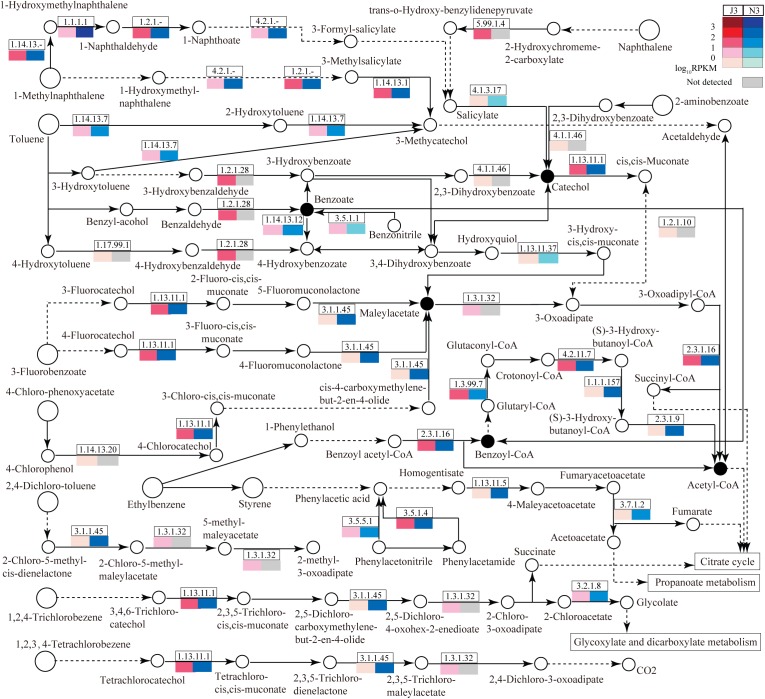
Relative abundances of enzymes related to the degradation of aromatic compounds in J3 and N3. A total of 11 abundant pathways associated with the degradation of aromatic compounds were analyzed: aminobenzoate degradation, benzoate degradation, fluorobenzoate degradation, chlorobenzene degradation, ethylbenzene degradation, naphthalene degradation, bisphenol degradation, styrene degradation, xylene degradation, polycyclic aromatic hydrocarbon degradation, and toluene degradation. In these pathways, only the portion of enzymes with relatively high expression levels is presented by EC number and total RPKM. Relative expression [log_10_RPKM)] is shown in red for J3 and blue for N3. The key products are highlighted with black closed circles.

As large differences of unigene numbers were found between J3 and N3 with regard to the degradation of aromatic compounds (Figure 1), this study also focused on DEGs related to the degradation of aromatic compounds; i.e., aminobenzoate degradation (ABD), benzoate degradation (BD), bisphenol degradation (BPD), chlorobenzene degradation (CBD), ethylbenzene degradation (EBD), fluorobenzoate degradation (FBD), naphthalene degradation (ND), polycyclic aromatic hydrocarbon degradation (PD), styrene degradation (SD), toluene degradation (TD), and xylene degradation (XD) (Figure 6 and Supplementary Table S9). Notably, pathways in degradation of aromatic compounds are unnecessary for microbes, and many enzymes have not previously been identified in these pathways. As shown in Figure 6, among these pathways, benzoate degradation (BD) serves as the key pathway to connect most of other pathways via its pivotal products, benzoate, benzoyl-coA, catechol, and maleylacetate. A portion of enzymes related to these pathways was detected in J3 and N3, many of which showed lower expression levels in J3 than in N3. In particular, large numbers of enzymes were detected in J3 that were not identified in N3. Moreover, some of these, i.e., 2-deoxy-D-gluconate 3-dehydrogenase (1.1.1.125), 2-hydroxychromene-2-carboxylate isomerase (5.99.1.4), benzaldehyde dehydrogenase (NAD) (1.2.1.28), D-3-phosphoglycerate dehydrogenase (1.1.1.95), NADPH2:quinone reductase (1.6.5.5), L-iditol 2-dehydrogenase (1.1.1.14), 5-carboxymethyl-2-hydroxymuconate isomerase (5.3.3.10), and aldehyde reductase (1.1.1.21) clearly showed relatively high transcript abundances, with RPKM values ranging from 9.8 to 102.0, in pathways of BD, ND, BPD, ABD, EBD, XD, and TD, respectively (Supplementary Table S9 and Figure 6). Highly expressed members of these enzymes in J3 mostly originated from fungal species, e.g., *N. fischeri*, *A. clavatus*, *Ajellomyces dermatitidis*, *Marssonina brunnea*, *Trichophyton equinum*, *Botryotinia fuckeliana*, and *A. oryzae* (Table 4). Therefore, it was considered reasonable to postulate that trace aromatic derivatives would be differently produced between J3 and N3, some of which might be only produced in J3. In addition, both J3 and N3 could degrade aromatic compounds into important end-products, such as acetyl-coA, fumarate, acetoacetate, succinate, and glycolate, which serve as intermediates for the citrate cycle, propanoate metabolism, and glycoxylate and dicarboxylate metabolism (Figure 6).

**Table 4:** The top 20 expressed enzymes for the degradation of aromatic compounds in J3.

Gene ID	K0 ID	EC ID	Definitions	RPKM	Species	Pathways
J3_701	K00011	1.1.1.21	Aldehyde reductase	101.4	*Aspergillus oryzae*	BD, ND, BPD
J3_3491	K00100	1.1.1.-	Dehydrogenase	55.0	*Paracoccidioides brasiliensis*	BD, ND, BPD
J3_1294	K00155	1.2.1.-	Dehydrogenase (NAD)	31.9	*Aspergillus oryzae*	ABD, ND, PD
J3_2545	K01826	5.3.3.10	5-carboxymethyl-2-hydroxymuconate isomerase	29.6	*Botryotinia fuckeliana*	BD
J3_3772	K00008	1.1.1.14	L-iditol 2-dehydrogenase	26.4	*Trichophyton equinum*	BD, ND, BPD
J3_787	K01113	3.1.3.1	Alkaline phosphatase D	26.1	*Aspergillus fumigatus*	ABD
J3_635	K00100	1.1.1.-	Dehydrogenase	21.1	*Penicillium marneffei*	BD, ND, BPD
J3_1295	K00100	1.1.1.-	Dehydrogenase	18.4	*Aspergillus terreus*	BD, ND, BPD
J3_20772	K00344	1.6.5.5	NADPH2:quinone reductase	16.6	*Marssonina brunnea*	ND
J3_3179	K00141	1.2.1.28	Benzaldehyde dehydrogenase (NAD)	16.1	*Aspergillus clavatus*	ABD, XD, TD
J3_124	K00058	1.1.1.95	D-3-phosphoglycerate dehydrogenase	16.0	*Ajellomyces dermatitidis*	BD
J3_2472	K00252	1.3.99.7	Glutaryl-CoA dehydrogenase	14.5	*Neosartorya fischeri*	BD
J3_988	K00517	1.14.-.-	Oxygenase	12.6	*Aspergillus niger*	ABD, BPD, PD
J3_300	K00632	2.3.1.16	Acetyl-CoA acyltransferase	12.5	*Aspergillus terreus*	BD, EBD
J3_1243	K00493	1.14.14.1	Unspecific monooxygenase	11.5	*Aspergillus oryzae*	ABD
J3_634	K14584	5.99.1.4	2-hydroxychromene-2-carboxylate isomerase	10.9	*Neosartorya fischeri*	ND
J3_1352	K03381	1.13.11.1	Catechol 1,2-dioxygenase	10.0	*Neosartorya fischeri*	BD, FBD, CBD, TD
J3_1961	K00065	1.1.1.125	2-deoxy-D-gluconate 3-dehydrogenase	9.3	*Neosartorya fischeri*	BD, ND, BPD
J3_4262	K01426	3.5.1.4	Amidase	9.2	*Penicillium chrysogenum*	ABD, SD
J3_246	K01692	4.2.1.17	Enoyl-CoA hydratase	8.5	*Aspergillus niger*	ABD, BD


*ABD, Aminobenzoate degradation; BD, benzoate degradation; BPD, bisphenol degradation; CBD, chlorobenzene degradation; EBD, ethylbenzene degradation; FBD, fluorobenzoate degradation; ND, naphthalene degradation; PD, polycyclic aromatic hydrocarbon degradation; SD, styrene degradation; TD, toluene degradation; XD, xylene degradation.*

## Discussion

The daqus of Chinese JF and NF liquor, the most consumed liquors in China, undergo markedly different production processes that make large contributions to their special flavors. To ascertain the underlying factors, in comparison with our previous work of NF daqu, the present study comprehensively revealed the active microbial community and enzymes at the high temperature stage (J3) of JF daqu, and comparatively analyzed the active enzyme profiles at high temperature stages of JF and NF daqus. The active fungal community produced more diverse enzymes than those of the bacterial community, with *Aspergillus* and *Penicillium* representing the dominant genera at J3. This finding was complementary to the previous microbial diversity revealed for JF daqu by 16S rRNA and ITS sequencing, which indicated that the bacterial community was more diverse than the fungal community at J3 (Huang et al., 2017b). Meanwhile, low abundances of active yeast might be due to high temperature condition at J3, which may be well consistent with previous finding that yeast decreased quickly from J2 (55°C) to J3 (70°C) (Huang et al., 2017b). Additionally, the prevailing role of the active fungal community was also revealed in NF daqu samples by metatranscriptomics analysis (Huang et al., 2017a). Therefore, the present study further confirmed the suitability of metatranscriptomics for obtaining the active microbial community profiles in daqus.

JF daqu exhibited lower numbers and expression levels of CAZymes at the high temperature stage of J3 than those at the high temperature stage of N3 (Huang et al., 2017a). In addition, except for the initial stage of J1, most CAZymes were detected with lower activities and less diversities in the production process of JF daqu samples than those of NF daqu samples (Table 2) (Huang et al., 2017a). Lower activities and diversities of amylases (one kind of CAZymes) were similarly found in JF than in NF daqu via activities assay and protein electrophoresis (Liu et al., 2018). Thus, these findings might to some extent be consistent with the lower capacities of saccharification and liquefaction in JF daqu than those in NF daqu (Liu and Sun, 2018; Liu et al., 2018). Additionally, several thermostable CAZymes were detected in special stages of JF daqu samples, such as α-amylase in J1 and J4, endo-β-1,3-1,4-glucanase in J3 and J4, endo-proteases in J2 and J4, and endo-1,4- β -D-xylanase in J4, which suggests the feasibility of mining thermostable enzymes from special stages in the future.

Based on the functional annotation, starch and sucrose metabolism was the most abundant pathway in J3, which might imply that the microbial community has full capacity for degrading different polymers into glucose in J3. Upon comprehensive comparison between J3 and N3, most pathways showed higher diversities with more unigenes in J3 than in N3, which indicated more complicated metabolism for the microbial community in J3. Moreover, large differences of diversities were observed in basic metabolisms, degradation of aromatic compounds, starch and sucrose metabolism, and amino sugar and nucleotide sugar metabolism between J3 and N3, which further suggested that the microbial community of J3 might produce higher diversities of metabolites, some of which, such as phenol, benzaldehyde, and phenylethanol, might serve as precursors for aroma compounds (Fan et al., 2012; Wang et al., 2014; Xiao et al., 2016). In contrast, similar diversities were found in oxidative phosphorylation and glycolysis between J3 and N3, which indicated that the microbial communities released considerable bio-heat to maintain the high temperatures of 62 and 70°C for several days in NF and JF daqu, respectively (Huang et al., 2017b). In addition, butanoate metabolism and pyruvate metabolism were also similarly active between J3 and N3, suggesting that their intermediates, such as butanoate and acetate, represented important substrates for flavor compounds of e.g., butanol, acetic acid, butanoic acid, ethyl hexanoate, hexyl acetate, and isopentyl butanoate in JF and NF liquor (Fan et al., 2012; Wang et al., 2014; Xiao et al., 2016). Furthermore, six amino acid metabolisms were dominant in both J3 and N3, the products of which, i.e., amino acids, a-keto acids, and aldehydes, may serve as pre-substrates for important flavor precursors such as pyrazine, alcohol, and acids (Badrinarayanan and Sperry, 2012; Nashalian and Yaylayan, 2014; Scalone et al., 2015; Xu et al., 2017). Therefore, the microbial community was both active at the high temperature stages of JF and NF daqu for generating bio-heat (Huang et al., 2017a,b; Xiao et al., 2017) and releasing flavor precursors (Wu et al., 2009; Zheng et al., 2011), and JF daqu could provide larger diversities of flavor precursors than NF daqu from most of the active pathways, in particular from the degradation of aromatic compounds.

Similar DEG profiles were observed between the high temperature stages of J3 and N3; thus, detailed functional comparisons of DEGs were performed between these stages with regard to four key carbohydrate and energy metabolisms: starch and sucrose metabolism, glycolysis, pyruvate metabolism, and the citrate cycle, as their intermediates are essential for ethanol and flavor generation. The results showed that both J3 and N3 contained an intact process for converting polymers into glucose, pyruvate, acetyl-coA, and ethanol, indicating a complete system for saccharification, liquefaction, and fermentation. In general, the majority of enzymes related to these four key pathways showed lower expression levels in J3 than in N3, indicating lower activities for enzymes in J3 than in N3 to a degree that is consistent with the lower capacities in saccharification, liquefaction, and fermentation exhibited by high-temperature JF daqu than those by medium-temperature NF daqu (Liu and Sun, 2018; Liu et al., 2018). Low expression levels of enzymes might result from the inhibition caused by the high temperature (70°C) in J3. However, some enzymes were only detected in J3, albeit with relative low expression levels, indicating that a large number of minor intermediates would likely be specifically generated in J3. Notably, among enzymes related to saccharification and liquefaction in J3, glucoamylases were clearly active with high expression levels, indicating their collaborative roles along with high temperature in degrading starches, which would be spontaneously decomposed under high temperature, as well as suggesting a feasible way to mine thermostable glucoamylases from J3. The majority of enzymes related to saccharification and liquefaction in J3 were highly expressed by fungal species of *R. emersonii*, *A. oryzae*, *A. fumigatus*, and *C. immitis*, some of which have been found to secrete numerous carbohydrate-active enzymes and show high capacities toward degrading polymers, such as *Aspergillus* (Culleton et al., 2013; de Vries et al., 2017; Cologna et al., 2018) and *R. emersonii* (Hua et al., 2014; Martínez et al., 2016). In addition, J3 showed considerable potential for converting glucose to pivotal intermediates, such as acetate, ethanol, pyruvate, and acetyl-coA, which might then serve as direct or indirect substrates for JF flavor compounds including ethyl acetate, ethyl butanoate, ethyl propanoate, ethyl 2-hydroxypropanoate, ethyl 2-hydroxyhexanoate, acetic acid, 2-acetylpyridine, hexyl acetate, benzyl acetate ethyl, ethyl 3-methylbutanoate, ethyl benzeneacetate, and 3-methylbutyl acetate (Fan et al., 2012; Wang et al., 2014; Xiao et al., 2016; Gao et al., 2017). The highly expressed enzymes related to glycolysis and pyruvate metabolism were mostly derived from fungal species, some of which have been applied to the production of fermented foods and drugs, such as *A. fumigatus* (Qin et al., 2012; Wakefield et al., 2017), *A. clavatus* (Mo et al., 2008; Zutz et al., 2013; Li et al., 2017), and *A. oryzae* (Park et al., 2018; Son et al., 2018; Zhong et al., 2018). Furthermore, low concentration of ethanol might be generated by several fungi in J3, which to some extent agreed with the earlier finding that a small amount of ethanol could be directly produced by co-culture of fungi (Takano and Hoshino, 2012). Additionally, relatively high expression levels of D-lactate dehydrogenase (cytochrome) might indicate high concentration of lactate in J3, which may be consistent with the high level of lactate in the subsequent mature JF daqu (Wu et al., 2009). Moreover, intermediates of the citrate cycle also serve as pre-substrates for flavor compounds, and the highly expressed enzymes related to this pathway also originated from the fungal community, some of which have been applied to the saccharification and fermentation process of foods and drugs, including *A. oryzae*, *A. clavatus*, and *A. terreus*.

Numerous aromatic compounds, such as tannin, ferulic acid, and lignin have been identified in the materials of cereals, the degradation of which is strongly related to liquor flavor generation (Liu and Sun, 2018). Several laccases, feruloyl esterase and ferulic acid decarboxylase were detected with low expression levels from *A. clavatus*, *C. posadasii*, *P. marneffei*, *A. terreus* or *Pseudomonas aeruginosa* in J3 (data not shown), which might clearly confirm the degradations of ferulic acid and lignin during high temperature stage of JF daqu. Similarly, many aromatic compounds and phenols were identified in both JF and NF liquors (Wang et al., 2014; Xiao et al., 2016); consistent with this, in the present study some enzymes related to the degradation of aromatic compounds were also found to be expressed in JF and NF liquor starters, with most showing lower expression in the former. However, the remainder constituted those enzymes that were only detected (at low levels) in J3, indicating that trace aroma compounds were likely particularly associated with JF liquor flavor, such as ethyl benzeneacetate and benzaldehyde (Xiao et al., 2016). Highly expressed members of these enzymes were mostly derived from fungal species in J3, which appears consistent with the contributions of some fungi toward the degradation of aromatic compounds (Godoy et al., 2016; Sun et al., 2016; Vieira et al., 2018). Alternatively, enzymes expressed at low levels from bacteria may also substantively contribute to degradation of aromatic compounds (Pérez-Pantoja et al., 2015; Van der Waals et al., 2017). Therefore, both the fungal and bacterial communities appear to have an active role in degrading aromatic compounds in JF daqu (Bhattacharya et al., 2017; Wang et al., 2017; Kamyabi et al., 2018; Ma et al., 2018), especially in the high temperature and mature stage (Huang et al., 2017b).

In addition to the microbial community, temperature also makes large contributions to generate flavor compounds in JF daqu, such as pyrazines and their derivatives, which comprise pivotal impact aroma compounds of JF liquor (Zhu et al., 2007; Fan et al., 2012). In particular, their generation may be thermally induced from microbial metabolites by non-enzymatic browning via the Maillard reaction at 70°C in J3 (Richards et al., 2011; Nashalian and Yaylayan, 2014). Overall, JF liquor flavor thus appears to be determined by a highly complicated process and further analysis of the active microbial community, enzymes, and metabolites from the daqu preparation in addition to stacking fermentation and alcoholic fermentation processes are required to unravel the mystery of JF liquor flavor generation.

## Conclusion

In the present study, fungi including *Aspergillus* and *Penicillium*, were identified as the most active microbial community members at the high temperature stage (J3: 70°C) of JF daqu by metatranscriptomics. Furthermore, the high temperature stage was found to not only lower the capacities of JF daqu toward saccharification and fermentation, but also enhance its ability in generating diverse minor flavor compounds, e.g., derivatives of aromatic compounds. Additionally, most of enzymes related to those capacities were highly expressed at 70°C by fungal genus of *Aspergillus*, *Coccidioides*, *Paracoccidioides*, *Penicillium*, and *Rasamsonia*. These exploratory findings shed light on our understanding of the JF baijiu fermentation system, in which the high temperature stage plays key roles in improving JF daqu by providing unique active microbiota and enzymes, and strongly contributing to the final distinctive aroma and taste of JF baijiu.

## Author Contributions

HZ, ZY, YF, YJ, LT, and KH designed the experiment. ZY performed the experiments and analyzed the data. ZY, YX, DL, and HL collected samples and communicated with the liquor factory. ZY and LC wrote the main manuscript. ZY, AD, YF, and HZ revised the manuscript. All authors revised and approved the final version of the manuscript.

## Conflict of Interest Statement

The authors declare that the research was conducted in the absence of any commercial or financial relationships that could be construed as a potential conflict of interest.

## Footnotes

1 http://trinityrnaseq.sourceforge.net/

2 https://www.kegg.jp/kegg/

3 http://wego.genomics.org.cn/cgi-bin/wego/index.pl

4 http://www.cazy.org/

5 http://weizhong-lab.ucsd.edu/cdhit_suite/cgi-bin/index.cgi
