# Supplementary material for: Unraveling the Contribution of High Temperature Stage to Jiang-Flavor Daqu, a Liquor Starter for Production of Chinese Jiang-Flavor Baijiu, With Special Reference to Metatranscriptomics
Source: Front Microbiol. 2019 Mar 12;10:472. doi: 10.3389/fmicb.2019.00472 (PMC6423406; doi:10.3389/fmicb.2019.00472)
Supplement: Supplementary file 1 [file Data_Sheet_1.docx]

Supplementary Material

Unraveling the contribution of high temperature stage to Jiang-flavor daqu, a liquor starter for production of Chinese Jiang-flavor baijiu, with special reference to metatranscriptomics

Zhuolin Yi^1,2,3^, Yanling Jin^2,3^, Yao Xiao^4^, Lanchai Chen^2,3,5^, Li Tan^2,3^, Anping Du^2,3^, Kaize He^2,3^, Dayu Liu^1^, Huibo Luo^6^, Yang Fang^2,3*^, Hai Zhao^1,2,3*^

*** Correspondence:**

Hai Zhao (email: zhaohai@cib.ac.cn) and Yang Fang (email: fangyang@cib.ac.cn), Environmental Microbiology Key Laboratory, Chengdu Institute of Biology, CAS, No.9 Section 4, Renmin Nan Road, Chengdu, 610041, Sichuan, P.R. China. Phone: +86 28 82890725; Fax: +86 28 82890733;

# Supplementary Figures

**
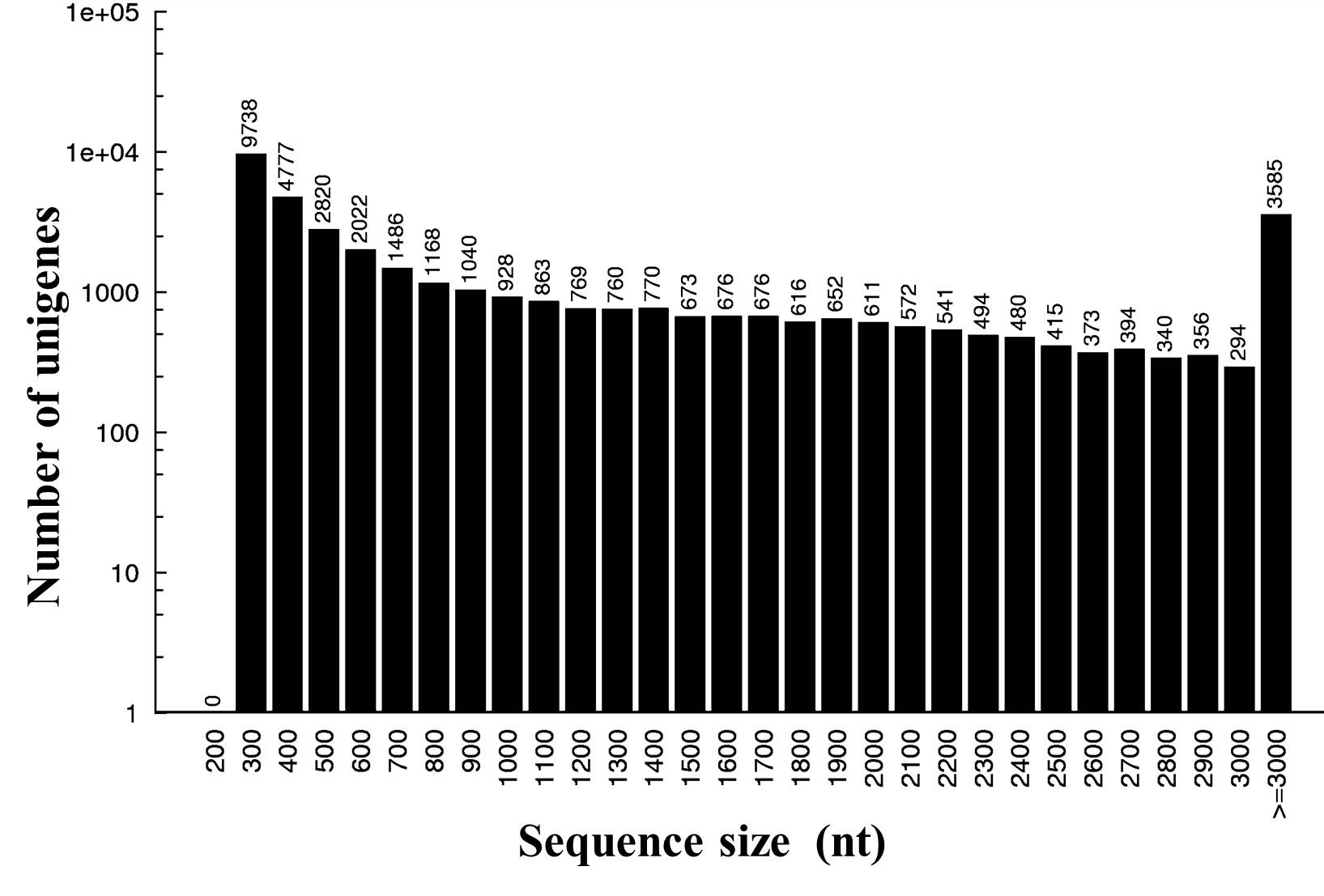
**

**Figure S1** The length distribution of all unigenes in J3.

**
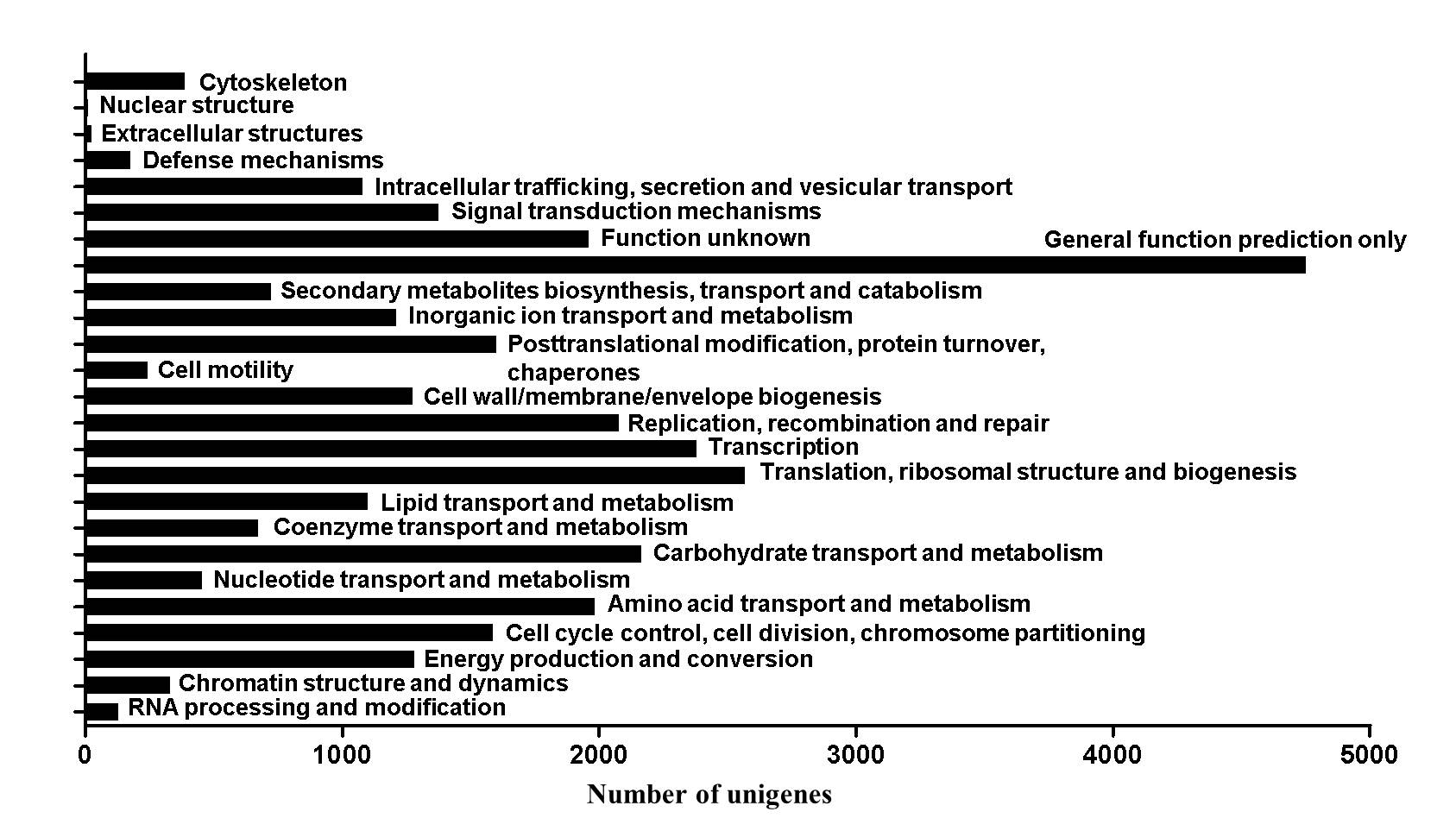
**

**Figure S2** COG functional classification of all unigenes in J3

**
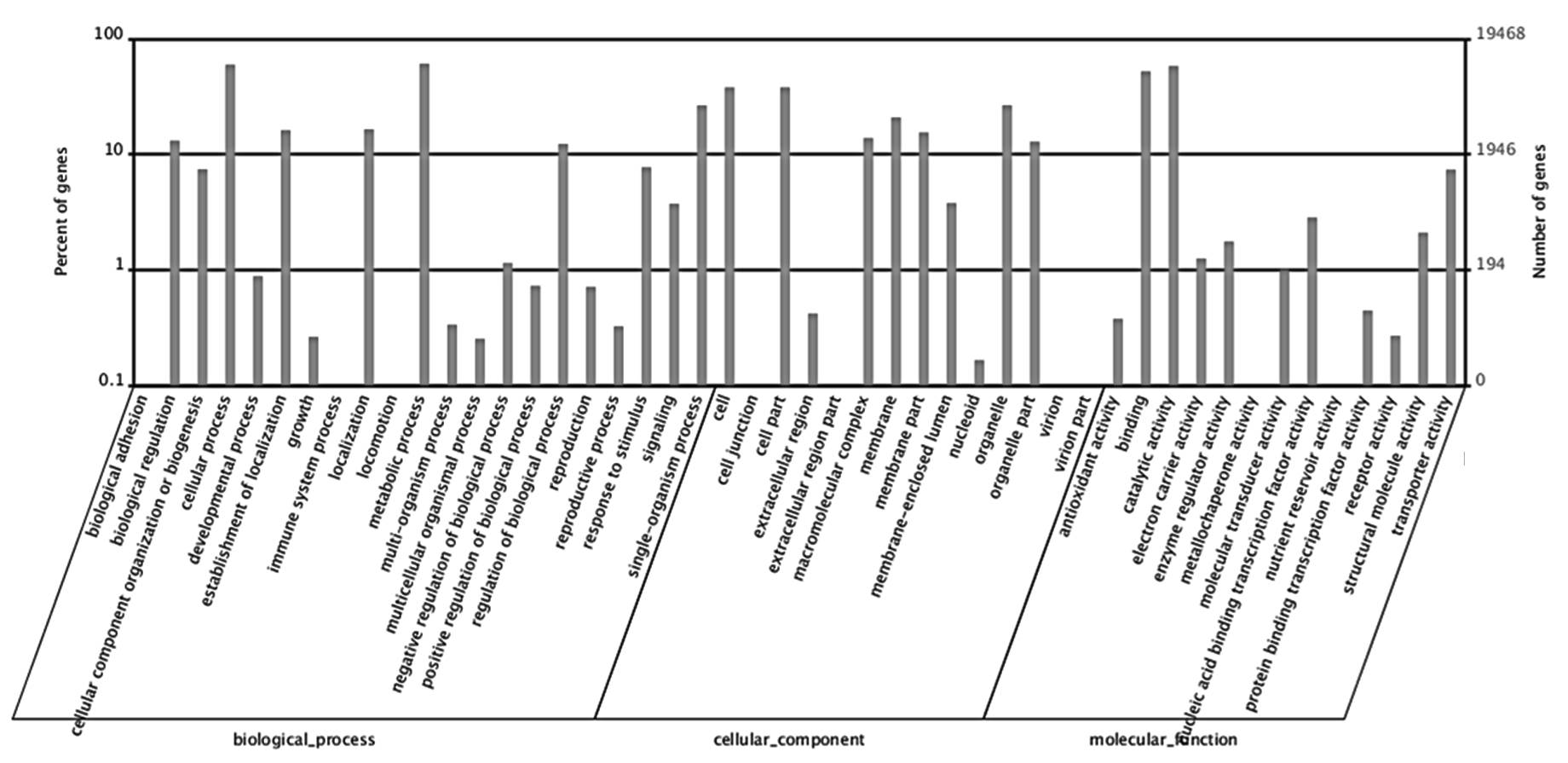
**

**Figure S3** GO functional classification of all unigenes in J3

**
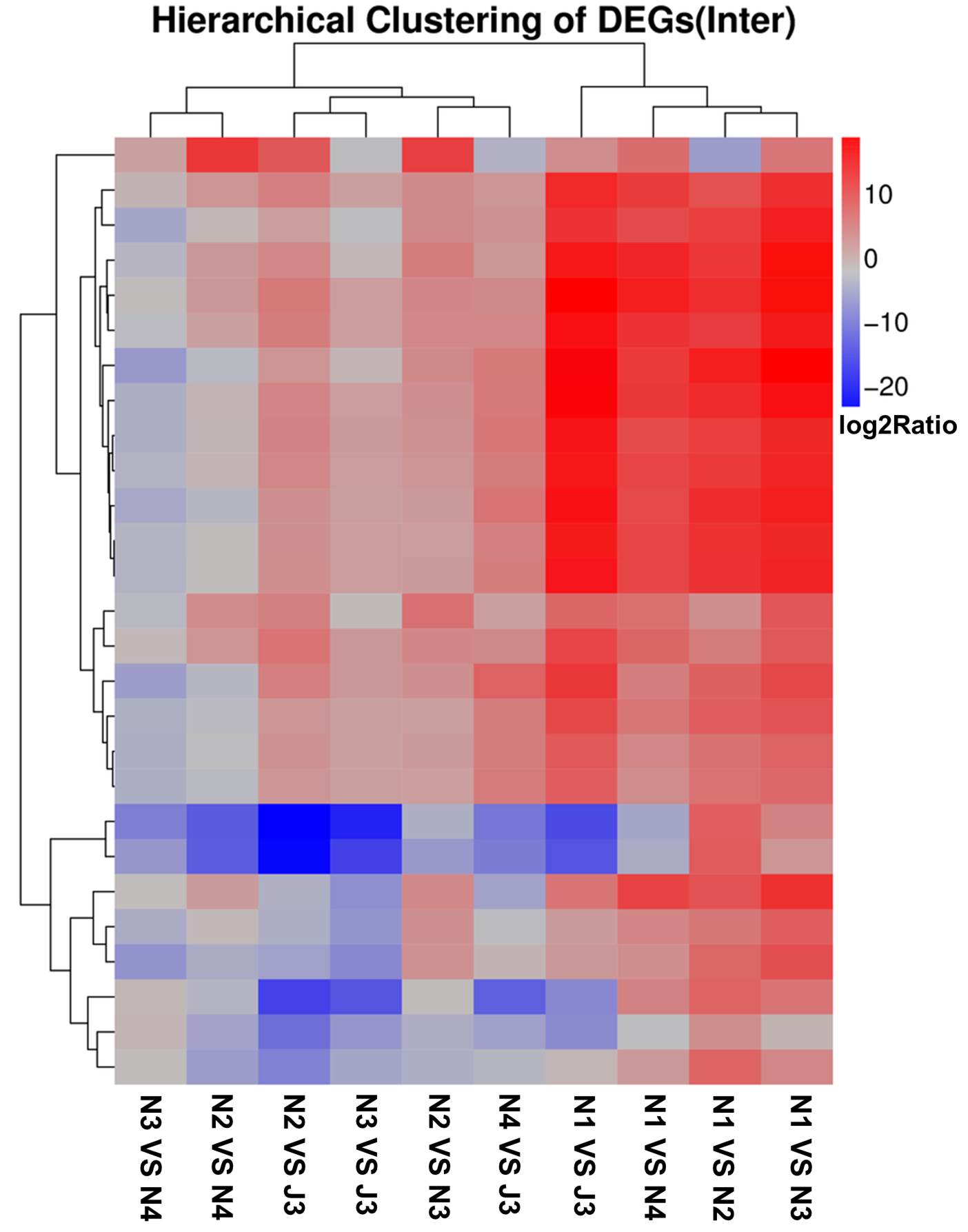
**

**Figure S4** Hierarchical clustering of differentially expressed genes (DEGs) (Inter) among J3 and NF samples (N1, N2, N3 and N4)


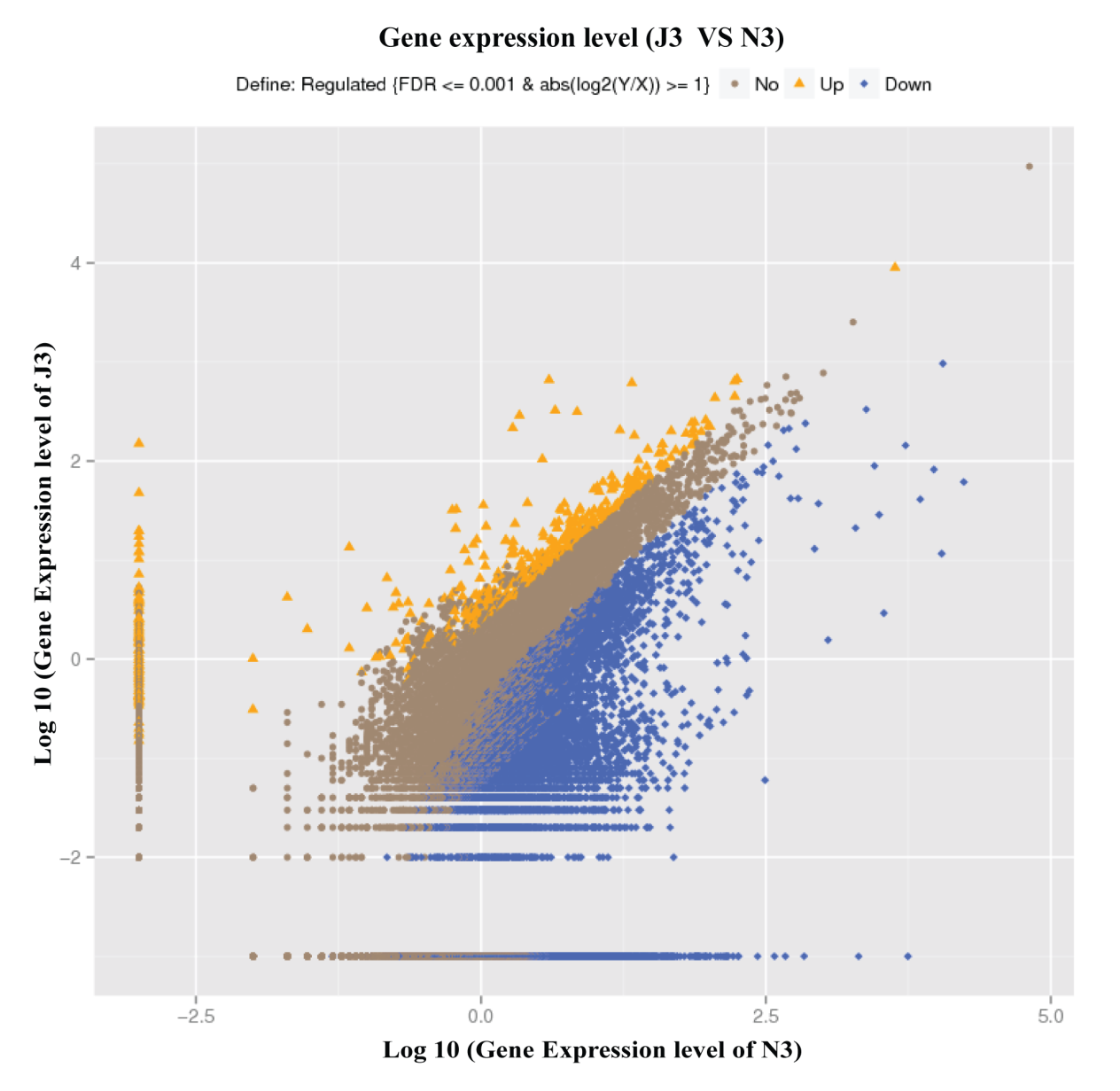


**Figure S5** Comparison of gene expression levels in J3 versus N3. Differentially expressed genes are indicated with orange triangle for up-regulation and blue diamond for down-regulation in J3 versus N3. Genes which were not differentially expressed between J3 and N3 are indicated with brown circle.

**
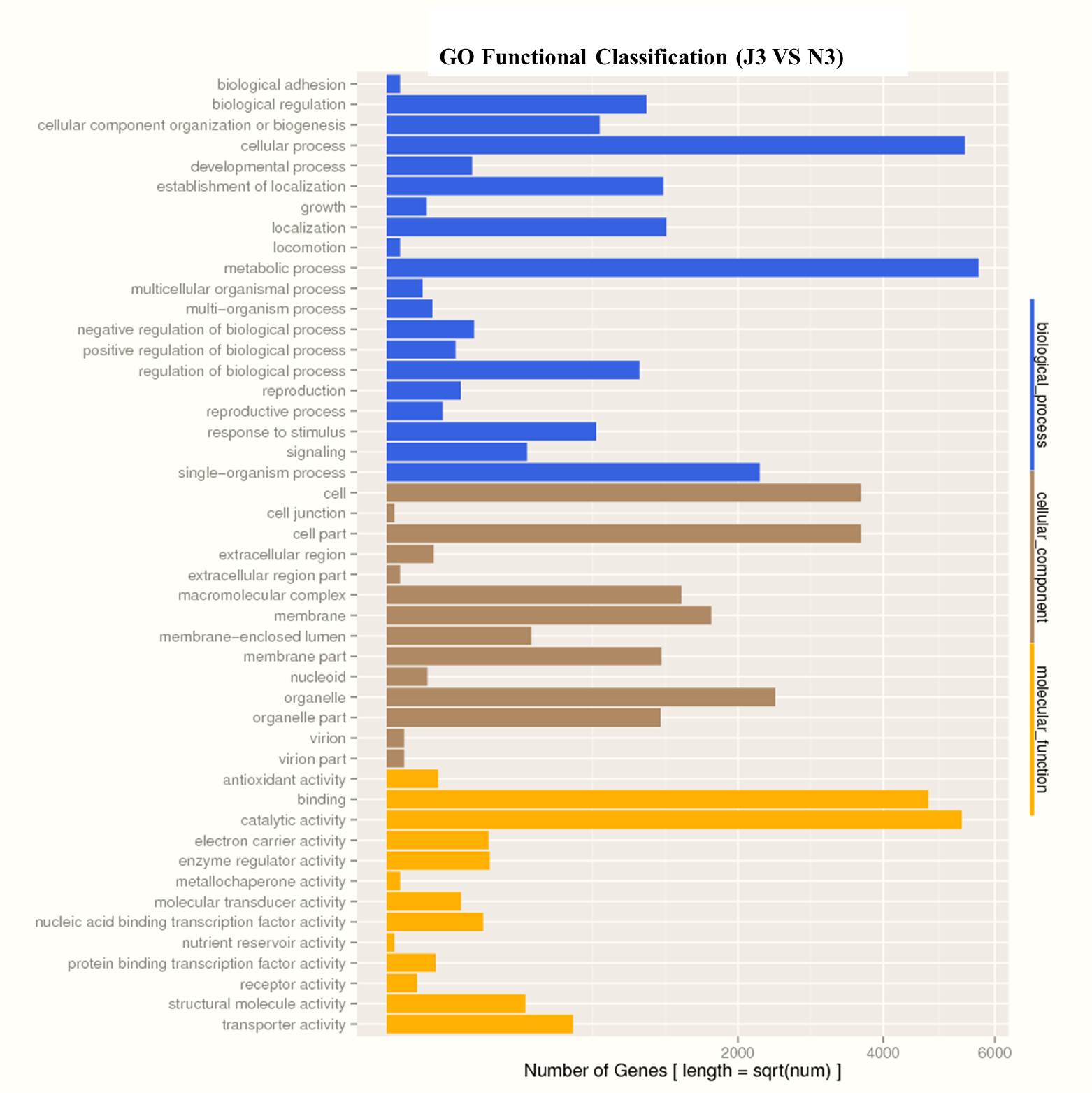
**

**Figure S6** GO functional classification of the DEGs in J3 versus N3

# Supplementary Tables

**Table S1** Raw RNA sequencing data and clean data statistic of J3 metatranscriptome.

| Sample ID | Raw Data (M) | Clean Data (M) | Data removed host/rRNA(bp) | Effective data rate (%) |
| --- | --- | --- | --- | --- |
| J3 | 5882 | 5663 | -- | 96.3 |

**Table S2** Assembly quantity statistics of J3 metatranscriptome.

|  | Sample ID | Total Number | Total Length (nt) | Mean Length (nt) | N50 | Distinct Clusters | Distinct Singletons |
| --- | --- | --- | --- | --- | --- | --- | --- |
| Contig | J3 | 106,462 | 35,507,250 | 334 | 821 | - | - |
| Unigene | J3 | 38,899 | 46,187,298 | 1188 | 2232 | 15621 | 23,268 |

**Table S3** Summary of annotation and CDs prediction of J3 metatranscriptome.

|  | Number of unigene hits | Percentagea |
| --- | --- | --- |
| All unigenes | 38,899 |  |
| All annotated unigenes | 31,279 | 80.4% |
| Annotated to NR database | 30,793 | 79.2% |
| Annotated to NT database | 20,894 | 53.7% |
| Annotated to Swiss-Prot database | 21,605 | 55.5% |
| Annotated to KEGG database | 21,736 | 55.9% |
| Annotated to COG database | 14,912 | 38.3% |
| Annotated to GO database | 19,468 | 50.1% |
| Total CDS | 31,654 | 81.4% |
| Mapped to the protein database | 30,613 | 78.7% |
| Predicted CDS | 1,041 | 2.7% |

**Table S4** The top 10 expressed carbohydrate-active enzyme families in J3 and their expressed levels in Nong-flavor liquor samples.

| Family | Relative expression (RPKM) | | | | |
| --- | --- | --- | --- | --- | --- |
|  | J3 | N1 | N2 | N3 | N4 |
| GH15 | 128.4 | nd | 226.0 | 153.1 | 95.9 |
| GH1 | 102.3 | 610.8 | 139.5 | 71.6 | 30.2 |
| GH18 | 67.6 | 1670.5 | 63.6 | 284.5 | 317.4 |
| GT2 | 44.3 | 1529.6 | 1763.3 | 1766.3 | 52.1 |
| GH28 | 28.7 | 271.0 | 1.7 | 152.5 | nd |
| GT20 | 28.2 | 308.8 | 1145.2 | 295.9 | 824.5 |
| GH79 | 20.4 | 27.8 | nd | 6.9 | nd |
| GT15 | 11.2 | nd | 186.7 | 512.9 | 175.0 |
| GH47 | 8.0 | 77.5 | 137.5 | 308.1 | 60.1 |
| GT57 | 7.7 | nd | 49.9 | 112.7 | nd |
| Others | 14.0 | 24589.8 | 12499.3 | 17125.4 | 4344.7 |
| Total | 460.8 | 29085.8 | 16212.8 | 20789.9 | 5899.9 |

nd: not determined.

**Table S5** The top 20 expressed enzymes related to Starch and sucrose metabolism in J3 and N3

| KO ID | EC ID | Definition | RPKM | |
| --- | --- | --- | --- | --- |
|  |  |  | J3 | N3 |
| K01178 | 3.2.1.3 | glucoamylase | 172.9 | 578.9 |
| K01835 | 5.4.2.2 | phosphoglucomutase | 83.6 | 433.9 |
| K01210 | 3.2.1.58 | glucan 1,3-beta-glucosidase | 70.1 | 372.3 |
| K01184 | 3.2.1.15 | polygalacturonase | 28.2 | 27.1 |
| K00697 | 2.4.1.15 | alpha,alpha-trehalose-phosphate synthase (UDP-forming) | 28.2 | 384.5 |
| K01179 | 3.2.1.4 | endoglucanase | 19.0 | 368.5 |
| K01188 | 3.2.1.21 | beta-glucosidase | 13.7 | 980.3 |
| K00844 | 2.7.1.1 | hexokinase | 7.3 | 396.3 |
| K01193 | 3.2.1.26 | beta-fructofuranosidase | 4.2 | nd |
| K00688 | 2.4.1.1 | starch phosphorylase | 2.4 | 13.5 |
| K01180 | 3.2.1.6 | endo-1,3(4)-beta-glucanase | 2.3 | 251.0 |
| K01187 | 3.2.1.20 | alpha-glucosidase | 1.8 | 761.7 |
| K00693 | 2.4.1.11 | glycogen(starch) synthase | 0.8 | 49.7 |
| K01213 | 3.2.1.67 | galacturan 1,4-alpha-galacturonidase | 0.5 | 6.9 |
| K01196 | 3.2.1.33 | glycogen debranching enzyme | 0.5 | 69.3 |
| K00706 | 2.4.1.34 | 1,3-beta-glucan synthase | 0.5 | 172.0 |
| K01199 | 3.2.1.39 | glucan endo-1,3-beta-D-glucosidase | 0.2 | 18.8 |
| K01194 | 3.2.1.28 | alpha,alpha-trehalase | 0.2 | 173.7 |
| K01176 | 3.2.1.1 | alpha-amylase | 0.2 | 812.3 |
| K15920 | 3.2.1.37 | beta-D-xylosidase | 0.1 | 184.8 |
| Others | | | 0.3 | 676.5 |

nd: not determined.

**Table S6** The top 20 expressed enzymes related to glycolysis pathway in J3

| KO ID | EC ID | Definitions | RPKM | |
| --- | --- | --- | --- | --- |
|  |  |  | J3 | N3 |
| K00128 | 1.2.1.3 | aldehyde dehydrogenase (NAD+) | 515.0 | 1300.4 |
| K00927 | 2.7.2.3 | phosphoglycerate kinase | 135.0 | 137.4 |
| K01835 | 5.4.2.2 | phosphoglucomutase | 83.6 | 433.9 |
| K00873 | 2.7.1.40 | pyruvate kinase | 75.5 | 269.4 |
| K01785 | 5.1.3.3 | aldose 1-epimerase | 19.9 | 277.0 |
| K01792 | 5.1.3.15 | glucose-6-phosphate 1-epimerase | 10.2 | 252.5 |
| K00844 | 2.7.1.1 | hexokinase | 7.5 | 360.7 |
| K00002 | 1.1.1.2 | alcohol dehydrogenase (NADP+) | 5.2 | 216.1 |
| K01803 | 5.3.1.1 | triosephosphate isomerase (TIM) | 4.3 | 255.3 |
| K15633 | 5.4.2.12 | 2,3-bisphosphoglycerate-independent phosphoglycerate mutase | 2.7 | nd |
| K13953 | 1.1.1.1 | alcohol dehydrogenase, propanol-preferring | 2.5 | 844.4 |
| K00627 | 2.3.1.12 | pyruvate dehydrogenase E2 component (dihydrolipoamide acetyltransferase) | 2.5 | 337.7 |
| K00001 | 1.1.1.1 | alcohol dehydrogenase | 2.0 | 522.2 |
| K00121 | 1.1.1.284 | S-(hydroxymethyl)glutathione dehydrogenase | 1.7 | 64.1 |
| K00134 | 1.2.1.12 | glyceraldehyde 3-phosphate dehydrogenase | 0.6 | 927.1 |
| K01568 | 4.1.1.1 | pyruvate decarboxylase | 0.4 | 1391.7 |
| K01610 | 4.1.1.49 | phosphoenolpyruvate carboxykinase (ATP) | 0.4 | 298.5 |
| K01689 | 4.2.1.11 | enolase | 0.3 | 544.7 |
| K00161 | 1.2.4.1 | pyruvate dehydrogenase E1 component subunit alpha | 0.3 | 308.6 |
| K01624 | 4.1.2.13 | fructose-bisphosphate aldolase, class II | 0.3 | 177.8 |
| Others | | | 0.9 | 2323.7 |

nd: not determined.

**Table S7** The top 20 expressed enzymes related to pyruvate metabolism in J3

| KO ID | EC ID | Definitions | RPKM | |
| --- | --- | --- | --- | --- |
|  |  |  | J3 | N3 |
| K00128 | 1.2.1.3 | aldehyde dehydrogenase (NAD+) | 515.0 | 1300.4 |
| K00102 | 1.1.2.4 | D-lactate dehydrogenase (cytochrome) | 162.1 | 439.6 |
| K01067 | 3.1.2.1 | acetyl-CoA hydrolase | 143.7 | 291.9 |
| K00011 | 1.1.1.21 | aldehyde reductase | 102.0 | nd |
| K00873 | 2.7.1.40 | pyruvate kinase | 75.5 | 269.4 |
| K01759 | 4.4.1.5 | lactoylglutathione lyase | 65.5 | 745.6 |
| K00026 | 1.1.1.37 | malate dehydrogenase | 37.8 | 849.5 |
| K00101 | 1.1.2.3 | L-lactate dehydrogenase (cytochrome) | 15.0 | 136.2 |
| K00627 | 2.3.1.12 | pyruvate dehydrogenase E2 component (dihydrolipoamide acetyltransferase) | 2.5 | 337.8 |
| K01649 | 2.3.3.13 | 2-isopropylmalate synthase | 1.3 | 149.1 |
| K00626 | 2.3.1.9 | acetyl-CoA C-acetyltransferase | 0.6 | 455.7 |
| K01512 | 3.6.1.7 | acylphosphatase | 0.4 | nd |
| K00467 | 1.13.12.4 | lactate 2-monooxygenase | 0.4 | 18.9 |
| K01610 | 4.1.1.49 | phosphoenolpyruvate carboxykinase (ATP) | 0.4 | 298.5 |
| K01069 | 3.1.2.6 | hydroxyacylglutathione hydrolase | 0.3 | 437.0 |
| K00161 | 1.2.4.1 | pyruvate dehydrogenase E1 component subunit alpha | 0.3 | 308.6 |
| K00162 | 1.2.4.1 | pyruvate dehydrogenase E1 component subunit beta | 0.2 | 339.4 |
| K01895 | 6.2.1.1 | acetyl-CoA synthase | 0.2 | 175.9 |
| K00382 | 1.8.1.4 | dihydrolipoamide dehydrogenase | 0.2 | 577.2 |
| K01638 | 2.3.3.9 | malate synthase | 0.1 | 290.3 |
| Others | | | 0.4 | 1200.0 |

nd: not determined.

**Table S8** The top 20 expressed enzymes related to citrate cycle pathway in J3

| KO ID | EC ID | Definitions | RPKM | |
| --- | --- | --- | --- | --- |
|  |  |  | J3 | N3 |
| K00030 | 1.1.1.41 | isocitrate dehydrogenase (NAD+) | 94.1 | 421.2 |
| K01679 | 4.2.1.2 | fumarate hydratase, class II | 81.3 | 300.7 |
| K00235 | 1.3.5.1 | succinate dehydrogenase (ubiquinone) iron-sulfur subunit | 63.0 | 375.8 |
| K00026 | 1.1.1.37 | malate dehydrogenase | 37.8 | 849.5 |
| K00658 | 2.3.1.61 | 2-oxoglutarate dehydrogenase E2 component | 7.9 | 238.2 |
| K01647 | 2.3.3.1 | citrate synthase | 4.9 | 337.9 |
| K01681 | 4.2.1.3 | aconitate hydratase 1 | 4.7 | 385.1 |
| K00627 | 2.3.1.12 | pyruvate dehydrogenase E2 component (dihydrolipoamide acetyltransferase) | 2.5 | 337.8 |
| K00244 | 1.3.99.1 | fumarate reductase flavoprotein subunit | 0.8 | nd |
| K01899 | 6.2.1.4 6.2.1.5 | succinyl-CoA synthetase alpha subunit | 0.7 | 144.1 |
| K01900 | 6.2.1.4 6.2.1.5 | succinyl-CoA synthetase beta subunit | 0.4 | 92.9 |
| K01610 | 4.1.1.49 | phosphoenolpyruvate carboxykinase (ATP) | 0.4 | 298.5 |
| K00031 | 1.1.1.42 | isocitrate dehydrogenase | 0.4 | 224.0 |
| K01648 | 2.3.3.8 | ATP citrate (pro-S)-lyase | 0.3 | 809.8 |
| K00161 | 1.2.4.1 | pyruvate dehydrogenase E1 component subunit alpha | 0.3 | 308.6 |
| K00236 | 1.3.5.1 | succinate dehydrogenase (ubiquinone) cytochrome b subunit | 0.2 | 287.4 |
| K00162 | 1.2.4.1 | pyruvate dehydrogenase E1 component subunit beta | 0.2 | 339.4 |
| K00382 | 1.8.1.4 | dihydrolipoamide dehydrogenase | 0.2 | 577.2 |
| K00234 | 1.3.5.1 | succinate dehydrogenase (ubiquinone) flavoprotein subunit | 0.2 | 464.5 |
| K00164 | 1.2.4.2 | 2-oxoglutarate dehydrogenase E1 component | 0.1 | 237.8 |
| Others | | | 0.2 | 1279.8 |

nd: not determined.

**Table S9** The relatively high expressed enzymes related to degradation of aromatic compounds in J3 and N3

| KO ID | EC ID | Definitions | RPKM | | Pathways |
| --- | --- | --- | --- | --- | --- |
|  |  |  | J3 | N3 |  |
| K00100 | 1.1.1.- | dehydrogenase | 128.8 | 1678.3 | BD, ND, BPD |
| K00011 | 1.1.1.21 | aldehyde reductase | 102.0 | nd | BD, ND, BPD |
| K00155 | 1.2.1.- | dehydrogenase (NAD) | 31.9 | 426.7 | ABD, ND, PD |
| K01826 | 5.3.3.10 | 5-carboxymethyl-2-hydroxymuconate isomerase | 29.6 | nd | BD |
| K01113 | 3.1.3.1 | alkaline phosphatase D | 28.4 | 61.9 | ABD |
| K00008 | 1.1.1.14 | L-iditol 2-dehydrogenase | 26.7 | nd | BD, ND, BPD |
| K00001 | 1.1.1.1 | alcohol dehydrogenase | 24.1 | 586.3 | BD, ND, BPD |
| K00344 | 1.6.5.5 | NADPH2:quinone reductase | 22.6 | nd | ND |
| K00924 | 2.7.1.- | kinase | 20.6 | nd | ABD, BD, EBD |
| K00480 | 1.14.13.1 | salicylate hydroxylase | 20.1 | 177.4 | ND,PD |
| K01426 | 3.5.1.4 | Amidase | 19.5 | 341.5 | ABD, SD |
| K00058 | 1.1.1.95 | D-3-phosphoglycerate dehydrogenase | 17.1 | nd | BD |
| K00141 | 1.2.1.28 | benzaldehyde dehydrogenase (NAD) | 16.4 | nd | ABD, XD, TD |
| K00252 | 1.3.99.7 | glutaryl-CoA dehydrogenase | 16.9 | 76.1 | BD |
| K01692 | 4.2.1.17 | enoyl-CoA hydratase | 15.6 | 469.9 | ABD, BD |
| K00632 | 2.3.1.16 | acetyl-CoA acyltransferase | 15.6 | 381.3 | BD, EBD |
| K00517 | 1.14.-.- | oxygenase | 14.6 | 633.7 | ABD, BPD,PD |
| K00493 | 1.14.14.1 | unspecific monooxygenase | 12.0 | 73.8 | ABD |
| K00492 | 1.14.13.- | oxygenase | 11.4 | 144.6 | ABD, CBD, ND, BPD |
| K03381 | 1.13.11.1 | catechol 1,2-dioxygenase | 11.3 | 223.1 | BD, FBD, CBD,TD |
| K14584 | 5.99.1.4 | 2-hydroxychromene-2-carboxylate isomerase | 10.9 | nd | ND |
| K00680 | 2.3.1.- | zearalenone synthase | 10.0 | 481.2 | ABD, BD, EBD, ND |
| K01066 | 3.1.1.- | esterase / lipase | 9.9 | 226.73 | ABD, BD,TD, PD |
| K00065 | 1.1.1.125 | 2-deoxy-D-gluconate 3-dehydrogenase | 9.8 | nd | BD, ND, BPD |
| K01501 | 3.5.5.1 | nitrilase | 8.6 | 14.2 | ABD, SD |
| K01560 | 3.8.1.2 | 2-haloacid dehalogenase | 6.9 | 76.2 | CBD |
| K01101 | 3.1.3.41 | 4-nitrophenyl phosphatase | 4.1 | 134.1 | ABD |
| K13953 | 1.1.1.1 | alcohol dehydrogenase, propanol-preferring | 2.5 | 799.7 | BD, ND |
| K01726 | 4.2.1.- | synthase | 2.4 | 316.7 | BD, ND, BPD |
| K00626 | 2.3.1.9 | acetyl-CoA C-acetyltransferase | 0.6 | 372.9 | BD |
| K01078 | 3.1.3.2 | acid phosphatase | 0.6 | 203.6 | ABD |
| K01061 | 3.1.1.45 | carboxymethylenebutenolidase | 0.4 | 356.5 | FBD, CBD, TD |
| K10437 | 1.14.13.- | phenylacetate 2-hydroxylase | 0.2 | 139.6 | ABD, BPD, SD |
| K00451 | 1.13.11.5 | homogentisate 1,2-dioxygenase | 0.1 | 783.9 | SD |
| K00074 | 1.1.1.157 | 3-hydroxybutyryl-CoA dehydrogenase | 0.1 | 356.5 | BD |
| K00257 | 1.3.99.- | dehydrogenase reductase | nd | 1900.6 | ND |
| K00599 | 2.1.1.- | methyltransferase | nd | 370.2 | PD |
| Others | | | 21.8 | 805.4 |  |

nd: not detected.

Abbreviation: Aminobenzoate degradation: ABD; Benzoate degradation: BD; Fluorobenzoate degradation: FBD; Chlorobenzene degradation: CBD; Ethylbenzene degradation: EBD; Naphthalene degradation: ND; Bisphenol degradation: BPD; Styrene degradation: SD; Xylene degradation: XD; Polycyclic aromatic hydrocarbon degradation: PD; Toluene degradation: TD.
